# A preliminary metagenomic and metabolomic investigation into the effects of *Aspergillus niger* cultures on microbial homeostasis and antibiotic resistance gene profiles in the rumen of fattening sheep

**DOI:** 10.1186/s40104-026-01412-z

**Published:** 2026-05-26

**Authors:** Yihan Wang, Yu Peng, Bo Wang, Meiqi Di, Minjie Xi, Zeyu Yao, Chengyu Shi, Qingchuan Feng, Dafei Yin, Jiantao Li, Xiangyi Xu, Ruiyang Zhang, Xianfeng Peng

**Affiliations:** 1 College of Animal Science and Veterinary Medicine, Shenyang Agricultural University, Shenyang, 110866 China; 2 Guangzhou Insighter Biotechnology Co., Ltd., Guangzhou, 510664 China

**Keywords:** Antibiotic resistance genes, *Aspergillus niger* cultures, High-concentrate diets, Metabolomics, Rumen microbiota

## Abstract

**Background:**

Under high-concentrate feeding conditions, ruminants often experience rumen microecological imbalance and dysfunction, which can impair growth performance and increase the risk of antibiotic resistance gene (ARG) dissemination.

**Results:**

To evaluate the ameliorative effects of *Aspergillus niger* (*A. niger*) cultures, fattening sheep were randomly allocated into the following five groups: a control group (CON), a control diet supplemented with 250, 500, or 1,000 mg/kg *A*. *niger* cultures (designated as LA, MA, and HA, respectively); and an antibiotic group supplemented with 5,000 mg/kg chlortetracycline premix (AN). Microbial community analysis indicated that several bacterial taxa, including *Succinivibrio* sp900317105, *Prevotella* sp002353485, *Quinella* sp017515635, *Quinella* sp015206805, and *Prevotella* sp900320255, were significantly enriched in the *A*. *niger* culture-supplemented groups (*P* < 0.05). ARG profiling showed that the abundance of tetracycline resistance genes was significantly lower in all *A. niger* groups compared with the CON and AN groups (*P* < 0.05), while β-lactam resistance genes were significantly reduced in the HA group (*P* < 0.05). Furthermore, the abundances of Rank I and Rank II ARGs were significantly higher in the AN group than in the other groups, whereas the abundances of Rank II and Rank IV ARGs were significantly lower in the *A. niger* culture groups than in the CON and AN groups. Metabolomic analysis further demonstrated that supplementation with *A. niger* cultures significantly decreased the concentration of N-decanoyl-L-homoserine lactone (*P* < 0.05) while increasing the levels of N-3-oxotetradec-7Z-enoyl-L-homoserine lactone, indole-3-methyl acetate, and indole-3-propionic acid (*P* < 0.05).

**Conclusions:**

These findings suggest that *A. niger* cultures can reduce the abundance of ARGs and mitigate the risk of ARG dissemination by modulating the rumen microbial community and associated metabolites.

**Graphical Abstract:**

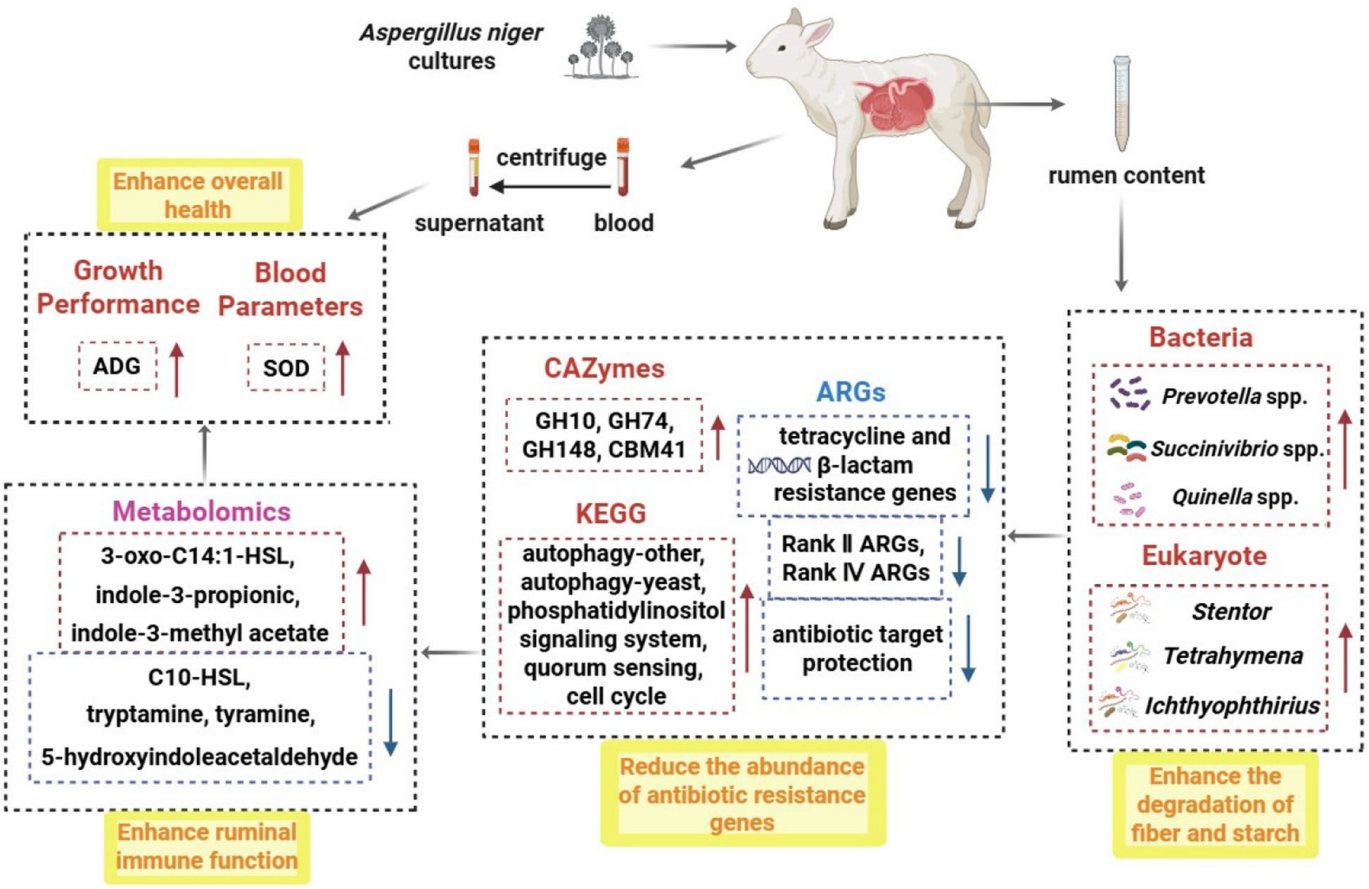

**Supplementary Information:**

The online version contains supplementary material available at 10.1186/s40104-026-01412-z.

## Introduction

With the rapid development of animal husbandry, enhancing rumen health and optimizing production performance have emerged as critical concerns in ruminant rearing [1]. Microbial feed additives, particularly fungal fermentation products such as *Aspergillus* spp., have been widely explored as effective strategies for enhancing rumen fermentation and metabolic efficiency, thereby improving growth performance, lactation productivity, and overall animal health [2, 3].

The genus *Aspergillus* is widely used as a microbial additive in ruminant nutrition. Among its species, *A. oryzae* has been extensively studied and has demonstrated significant benefits for growth performance, rumen health, and microbial homeostasis [4]. Similar to *A. oryzae*, *A. niger* and its fermentation cultures are rich in extracellular enzymes (including cellulase, amylase, and pectinase), metabolites, and secondary metabolites [5]. These characteristics provide *A. niger* and its cultures with considerable potential to enhance rumen microbial balance and improve nutrient utilization in ruminants. Previous studies have provided preliminary evidence supporting the beneficial effects of *A*. *niger*. For example, in vitro rumen fermentation experiments showed that supplementation with *A. niger* cultures significantly increased gas production and exhibited strong amylolytic activity [6]. In addition, extracts obtained from the co-cultivation of *A. oryzae* and *A. niger* have been reported to significantly improve in vitro digestibility, rumen fermentation characteristics, and bacterial community composition [7]. However, despite these promising findings, systematic in vivo studies investigating the effects of *A. niger* cultures on ruminant physiology and rumen microbial ecology remain limited.

Antimicrobial resistance (AMR) represents a pressing global public health challenge, significantly jeopardizing antibiotic efficacy and elevating the rates of treatment failure and mortality in both humans and animals [8]. The rumen, as a complex microbial ecosystem, has been identified as a major reservoir of antibiotic resistance genes (ARGs) [9]. These genes can persist within the rumen microbiota and may enter environmental systems through animal excretion. Moreover, ARGs have the potential to disseminate to humans via the food chain or direct contact with livestock, thereby amplifying public health risks associated with antimicrobial resistance [10]. Previous studies have revealed that nutritional regulation measures can influence the abundance and distribution of ARGs by altering the composition of rumen microbiota [9]. Given the potential of *A. niger* cultures to regulate rumen microbiota, it is important to determine whether such supplementation can also reduce the risk of ARG dissemination. Therefore, this study preliminarily and systematically investigated the effects of *A. niger* cultures on growth performance, rumen microbial composition, ARGs, and metabolic profiles in fattening sheep. The results are anticipated to contribute to the scientific basis for applying *A. niger* cultures as functional feed additives in ruminant production systems.

## Materials and methods

### Experimental design

In this study, 50 fattening female lambs derived from crossbreeding between Small-tailed and Charolais sheep (14.60 ± 0.22 kg body weight, BW) at 3 months of age were selected and reared in 2 m × 2 m enclosures at the experimental station of Shenyang Agricultural University. All sheep were fed an identical diet without *A. niger* for a 14-day pre-feeding period. The experimental sheep were randomly allocated into five treatment groups and fed the following diets: a control diet (CON; *n* = 10), a control diet supplemented with 250 mg/kg *A. niger* cultures (LA; *n* = 10), a control diet supplemented with 500 mg/kg *A. niger* cultures (MA; *n* = 10), a control diet supplemented with 1,000 mg/kg *A. niger* cultures (HA; *n* = 10), and a control diet supplemented with 5,000 mg/kg chlortetracycline premix (AN; *n* = 10). The chlortetracycline premix (Citifac, CP BIO) contained 15% chlortetracycline. The formulation of the daily rations was according to NY/T 816–2021 [11], and the composition and nutritional analysis of the control diet are presented in Table S1. The experimental period lasted 42 d. The experimental sheep were maintained in individual pens, with water available ad libitum. The animals were fed a total mixed ration twice daily at 0800 h and 1700 h. Throughout the experiment, daily feed intake was recorded for each sheep, and BW was measured weekly before the morning feeding to calculate initial body weight (IBW), final body weight (FBW), average daily gain (ADG), average daily feed intake (ADFI) and feed-to-gain ratio (F/G).

### Sample collection

At the end of the experimental period (d 42), rumen contents and blood samples were collected in the morning before feeding from eight randomly selected sheep per group. Rumen fluid was collected using a rumen tube [12], and the rumen contents were filtered through four layers of cheesecloth [13]. The ruminal pH was then measured using a glass electrode (pH400, Analysis Instrument Technology Co., Ltd., Shanghai, China). The remaining samples were stored at −80 °C for subsequent analyses, including fermentation parameters, microbial composition, and metabolomic profiling. On d 42, blood samples were collected via jugular venipuncture from the experimental sheep, and the separated plasma supernatants were stored at −80 °C for further analysis.

### Blood biochemical, antioxidant and immunological indicator measurement

Total protein (TP), albumin (ALB), globulin (GLB), total cholesterol (TC), triglycerides (TG), alanine aminotransferase (ALT), aspartate aminotransferase (AST), lactate dehydrogenase (LDH), alkaline phosphatase (ALP), glucose (GLU), high-density lipoprotein (HDL), low-density lipoprotein (LDL), and blood urea nitrogen (BUN) were determined using a fully automated biochemical analyzer (BS-420, Myriad Biomedical Electronics Co., Ltd., Shenzhen, China). In addition, glutathione peroxidase (GSH-Px), superoxide dismutase (SOD), malondialdehyde (MDA), catalase (CAT) and total antioxidant capacity (T-AOC) were measured using commercial assay kits according to the manufacturer’s instructions (HY-60021, Huaying Institute of Biotechnology, Beijing, China). Moreover, the levels of immunoglobulin A (IgA), immunoglobulin G (IgG), and immunoglobulin M (IgM) were determined according to previously reported methods [14].

### Rumen fermentation parameters

The concentrations of volatile fatty acids (VFA) and ammonia nitrogen (NH_3_-N) in rumen contents were detected using a gas chromatograph (GC-7890B, Agilent Technologies, Shanghai, China) and a spectrophotometer (Model 722S, Shanghai Prism Technology Co., Ltd., Shanghai, China), respectively, following the procedures described by Hu et al. [15]. Furthermore, according to the method reported by Ge et al. [16], the Caulmers Brilliant Blue method and enzyme labeling (Infinite M200 pro, TECAN, Inc., Zurich, Switzerland) assay techniques were employed for the detection of microbial protein (MCP) concentrations.

### Rumen microbial DNA extraction, metagenomic sequencing, and data analysis

Total DNA was extracted from sheep rumen content samples using the E.Z.N.A.^®^ Stool DNA Kit (Omega Bio-tek, Norcross, GA, USA) according to the manufacturer’s instructions. Sequencing libraries were constructed from high-quality DNA samples (OD260/280 = 1.8 to 2.2, OD260/230 ≥ 2.0). Metagenomic sequencing libraries for each sample were prepared using the TruSeq DNA Library Preparation Kit (Catalog No. FC-121-2001, Illumina, USA). All libraries were quantified and subsequently sequenced by Shanghai Bazelon Biotechnology Co., Ltd. All samples were sequenced in double-ended 150 bp (PE150) mode on a next-generation sequencing (NGS) platform. Diversity indices, including richness, Simpson, and Shannon indices, were calculated using Mothur v.1.21.1 [17]. Beta diversity was assessed using the Community Ecology R-vegan software package (version 2.6–4). The raw sequencing data have been deposited in the NCBI Sequence Read Archive under accession number PRJNA1345050.

### Rumen metabolome

Separation of polar metabolites was achieved using a Waters ACQUITY UPLC BEH Amide column (2.1 mm × 50 mm, 1.7 μm) [18] installed on a Vanquish UPLC system (Thermo Fisher Scientific, Massachusetts, USA). The mobile phase was composed of A: an aqueous solution of 25 mmol/L ammonium acetate and 25 mmol/L ammonia, and B: acetonitrile. The sample tray temperature was set at 4 °C, and a 2-μL sample was injected. Mass spectrometry data, both primary and secondary, were collected on an Orbitrap Exploris 120 instrument controlled by the software (Xcalibur, version 4.4, Thermo).

Following the conversion of raw data to mzXML format using ProteoWizard, metabolites were identified via the collaborative R package in conjunction with the BiotreeDB (v3.0) database [19]. Partial least squares discriminant analysis (PLS-DA) and orthogonal partial least squares discriminant analysis (OPLS-DA) of the metabolomic data were conducted using SIMCA-P 13.0 (Umetrics, Umeå, Sweden).

### Statistical analysis

All data, including growth performance, blood parameters, rumen fermentation parameters, and microbial diversity indices, were subjected to one-way ANOVA using SPSS software (version 22.0, SPSS Inc., Chicago, IL, USA), and multiple comparisons were performed using Duncan’s test. Specifically, only the abundant microbial taxa at the phylum and genus levels with an abundance greater than 0.1% were included in the statistical analysis, as described by Zhang et al. [20]. Results are presented as mean ± standard error of the mean (SEM). *P* ≤ 0.05 was considered statistically significant.

Principal coordinate analysis (PCoA) of microbial composition and functions was performed using the R-vegan software package (version 4.3.2). Gephi 0.8.2 (https://gephi.org/) was applied to conduct the network diagrams. Differential metabolites were identified through statistical analysis combined with variable importance in the projection (VIP, generated from PLS-DA analysis) values. Ruminal metabolites meeting the criteria of *P* < 0.05 and VIP > 1 were defined as differential metabolites between the two dietary treatments.

## Results

### Growth performance

In terms of growth performance, the groups did not differ significantly in the IBW, FBW, and ADFI of fattening sheep (*P* > 0.05). Compared with the CON group, a markedly higher ADG (*P* < 0.05) was observed in the AN and HA groups. Throughout the experimental period, the HA group had the numerically lowest F/G, followed by the AN group, but the groups did not differ significantly (*P* > 0.05, Table 1).

**Table 1:** The effects of dietary treatments on growth performance of fattening sheep

Item^1^	Groups^2^					SEM^3^	*P*-value
	**CON**	**AN**	**LA**	**MA**	**HA**		
IBW, kg	14.98	14.35	14.76	14.56	14.33	0.219	0.873
FBW, kg	22.16	23.28	21.69	21.91	22.63	0.257	0.307
ADG, g/d	170.88^c^	212.88^a^	164.25^c^	175.25^bc^	197.63^ab^	4.528	0.001
ADFI, g	1,079.5	1,270.13	1,096	1,128.63	1,146.5	29.422	0.270
F/G	6.36	5.98	6.75	6.46	5.87	0.156	0.382

^1^*IBW* Initial body weight, *FBW* Final body weight, *ADG* Average daily gain, *ADFI* Average daily feed intake, *F/G* Feed to gain ratio

^2^CON = a control diet; AN = a control diet + 5,000 mg/kg chlortetracycline premix; LA = a control diet + 250 mg/kg *A. niger* cultures; MA = a control diet + 500 mg/kg *A. niger* cultures; HA = a control diet + 1,000 mg/kg *A. niger* cultures

^3^Standard error of the mean

^a–c^Within the same row, means with different superscripts indicate statistically significant differences (*P* < 0.05)

### Blood biochemical, antioxidant, and immunological indicators

As far as blood biochemical indices were concerned, compared with the AN, a marked increase in TP content was observed in the LA and MA groups (*P* < 0.05). The *A. niger* culture groups exhibited significantly elevated GLB content compared with the AN group (*P* < 0.05). In addition, compared with the MA group, GLU content was higher in the LA, AN, and CON groups (*P* < 0.05). No significant effects of the dietary treatments were observed on the remaining blood biochemical indices (*P* > 0.05; Table 2).

**Table 2:** The effects of dietary treatments on blood biochemical, antioxidant and immunological indices in fattening sheep

Item^1^	Groups^2^					SEM^3^	*P*-value
	**CON**	**AN**	**LA**	**MA**	**HA**		
TP, g/L	61.73^ab^	58.76^b^	63.68^a^	65.70^a^	61.67^ab^	0.713	0.023
ALB, g/L	28.93	30.21	29.58	28.15	28.01	0.393	0.351
GLB, g/L	32.80^ab^	28.56^b^	34.11^a^	37.55^a^	33.66^a^	0.852	0.014
TC, mmol/L	1.26	1.34	1.30	1.41	1.22	0.040	0.647
TG, mmol/L	0.29	0.27	0.37	0.33	0.30	0.023	0.658
HDL, mmol/L	0.56	0.63	0.61	0.63	0.60	0.017	0.746
LDL, mmol/L	0.50	0.46	0.46	0.56	0.41	0.027	0.497
GLU, mmol/L	4.59^a^	5.07^a^	4.89^a^	3.93^b^	4.54^ab^	0.110	0.007
BUN, mg/dL	15.89	16.78	16.49	19.00	17.12	0.492	0.343
AST, U/L	119.85	143.66	148.11	131.99	145.63	5.568	0.481
ALT, U/L	22.07	29.64	24.57	25.31	26.20	1.470	0.612
ALP, U/L	503.70	592.81	501.83	366.18	474.72	25.823	0.085
LDH, U/L	536.09	626.33	624.90	551.52	607.30	14.817	0.156
SOD, U/mL	67.41^b^	71.98^ab^	65.64^b^	80.51^a^	80.99^a^	1.962	0.021
GSH-PX, U/mL	145.78	164.20	152.81	158.39	166.66	4.014	0.482
T-AOC, U/mL	6.32	6.75	6.20	6.70	7.60	0.182	0.115
CAT, U/mL	30.81	34.84	32.28	35.53	36.95	0.876	0.163
MDA, nmol/mL	5.07	4.55	5.48	4.20	3.76	0.207	0.060
IgA, g/L	1.39	1.28	1.34	1.40	1.40	0.029	0.635
IgG, g/L	20.44	19.68	21.02	21.93	21.33	0.383	0.417
IgM, g/L	0.85	0.79	0.86	0.9	0.88	0.018	0.386

^1^*TP* Total protein, *ALB* Albumin, *GLB* Globulin, *TC* Total cholesterol, *TG* Triglycerides, *HDL* High-density lipoprotein, *LDL* Low-density lipoprotein, *GLU* Glucose, *BUN* Blood urea nitrogen, *AST* Aspartate aminotransferase, *ALT* Alanine aminotransferase, *ALP* Alkaline phosphatase, *LDH* Lactate dehydrogenase, *SOD* Superoxide dismutase, *GSH-PX* Glutathione peroxidase, *T-AOC* Total antioxidant capacity, *CAT* Catalase, *MDA* Malondialdehyde, *IgA* Immunoglobulin A, *IgG* Immunoglobulin G, *IgM* Immunoglobulin M

^2^CON = a control diet; AN = a control diet + 5,000 mg/kg chlortetracycline premix; LA = a control diet + 250 mg/kg *A. niger* cultures; MA = a control diet + 500 mg/kg *A. niger* cultures; HA = a control diet + 1,000 mg/kg *A. niger* cultures

^3^Standard error of the mean

^a,b^Means with different superscripts indicate statistically significant differences within corresponding sampling times and variables (*P* < 0.05)

Moreover, compared with the CON and LA groups, the MA and HA groups displayed a significant elevation in SOD levels (*P* < 0.05); these levels were comparable to those in the AN (*P* > 0.05). Dietary supplementation did not significantly (*P* > 0.05) affect GSH-PX, T-AOC, CAT, and MDA contents in blood antioxidant indices in fattening sheep. Also, there was no significant effect on IgA, IgG, and IgM contents in blood immunity indexes (*P* > 0.05; Table 2).

### Ruminal pH and fermentation parameters

As shown in Table 3, compared with the CON, LA, and MA groups, a significant elevation (*P* < 0.05) in ruminal pH was observed in the AN group of fattening sheep. However, the HA group exhibited no marked difference (*P* > 0.05) in ruminal pH compared with all remaining groups. None of the rumen fermentation parameters (acetate, propionate, butyrate, valerate, MCP, and NH_3_-N) were markedly influenced (*P* > 0.05) by the dietary treatments (Table 3).

**Table 3:** The effects of dietary treatments on rumen pH and fermentation parameters in fattening sheep

Item^1^	Groups^2^					SEM^3^	*P*-value
	**CON**	**AN**	**LA**	**MA**	**HA**		
pH	6.95^b^	7.33^a^	6.83^b^	7.00^b^	7.12^ab^	0.050	0.017
Acetate, mmol/L	42.40	43.98	49.17	48.36	57.73	2.672	0.418
Propionate, mmol/L	11.89	11.31	17.24	13.41	17.14	1.045	0.208
Butyrate, mmol/L	10.72	7.88	9.71	9.94	14.62	0.916	0.206
Valerate, mmol/L	0.46	0.26	0.54	0.37	0.58	0.041	0.096
Isobutyrate, mmol/L	0.34	0.09	0.46	0.28	0.09	0.059	0.197
Isovalerate, mmol/L	0.41	0.65	0.61	0.63	0.77	0.055	0.37
TVFA, mmol/L	66.22	64.18	77.73	72.98	90.92	4.429	0.332
MCP, mg/dL	19.17	18.8	19.08	19.68	19.39	0.485	0.987
NH_3_-N, mg/dL	11.42	12.08	11.93	12.44	13.04	0.377	0.742

^1^*TVFA* Total volatile fatty acids, *MCP* Microbial protein

^2^CON = a control diet; AN = a control diet + 5,000 mg/kg chlortetracycline premix; LA = a control diet + 250 mg/kg *A. niger* cultures; MA = a control diet + 500 mg/kg *A. niger* cultures; HA = a control diet + 1,000 mg/kg *A. niger* cultures

^3^Standard error of the mean

^a,b^Within the same row, means with different superscripts indicate statistically significant differences (*P* < 0.05)

### Rumen microbial diversity

**Fig. 1:**
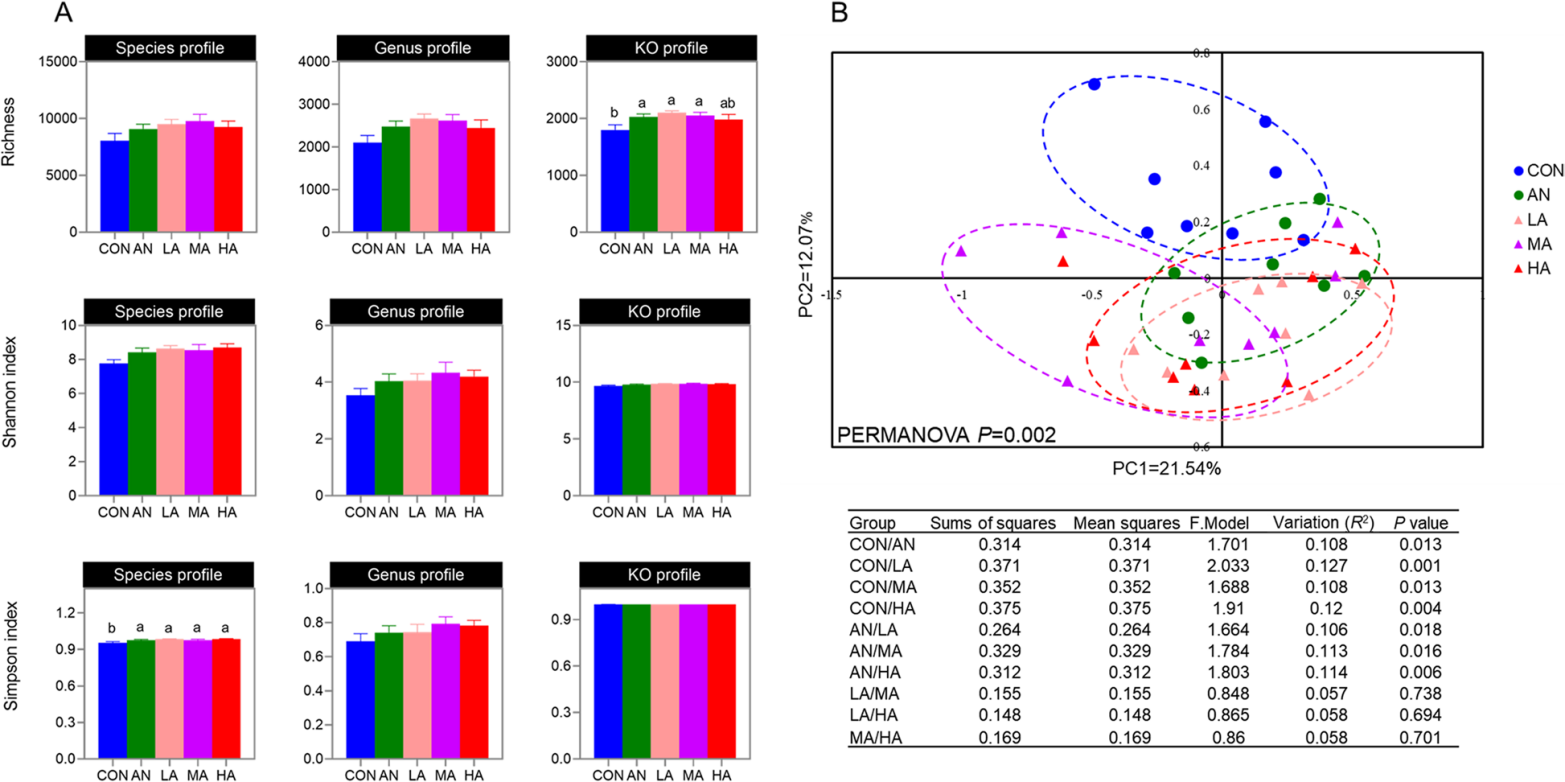
Rumen microbial diversity of fattening sheep. **A** The richness, Shannon and Simpson indices of rumen microbiota. **B** PCoA and PERMANOVA of rumen microbiota. CON = a control diet; AN = a control diet + 5,000 mg/kg chlortetracycline premix; LA = a control diet + 250 mg/kg *A*. *niger* cultures; MA = a control diet + 500 mg/kg* A*. *niger* cultures; HA = a control diet + 1,000 mg/kg *A*. *niger* cultures. ^a,b^Different superscripts indicate statistically significant differences (*P* < 0.05)

The effects of different ration treatments on ruminal microbial diversity in fattening sheep are shown in Fig. 1A. The analysis demonstrated a significantly elevated (*P* < 0.05) rumen microbial KO abundance richness index in the AN, LA, and MA groups compared with the CON group. Moreover, compared with the CON group, all four experimental groups harbored a significantly elevated (*P* < 0.05) Simpson index in the rumen microbiota at the species level. No significant effects of the different ration treatments were observed on the other diversity indices (*P* > 0.05). The PCoA and PERMANOVA results indicated that the rumen microbiota of the CON and AN groups were significantly distinct (*P* < 0.05) from those of the *A. niger* culture groups (Fig. 1B). In contrast, no significant differences were observed among the different *A. niger* culture groups (*P* > 0.05).

### Composition of the rumen bacteria

**Fig. 2:**
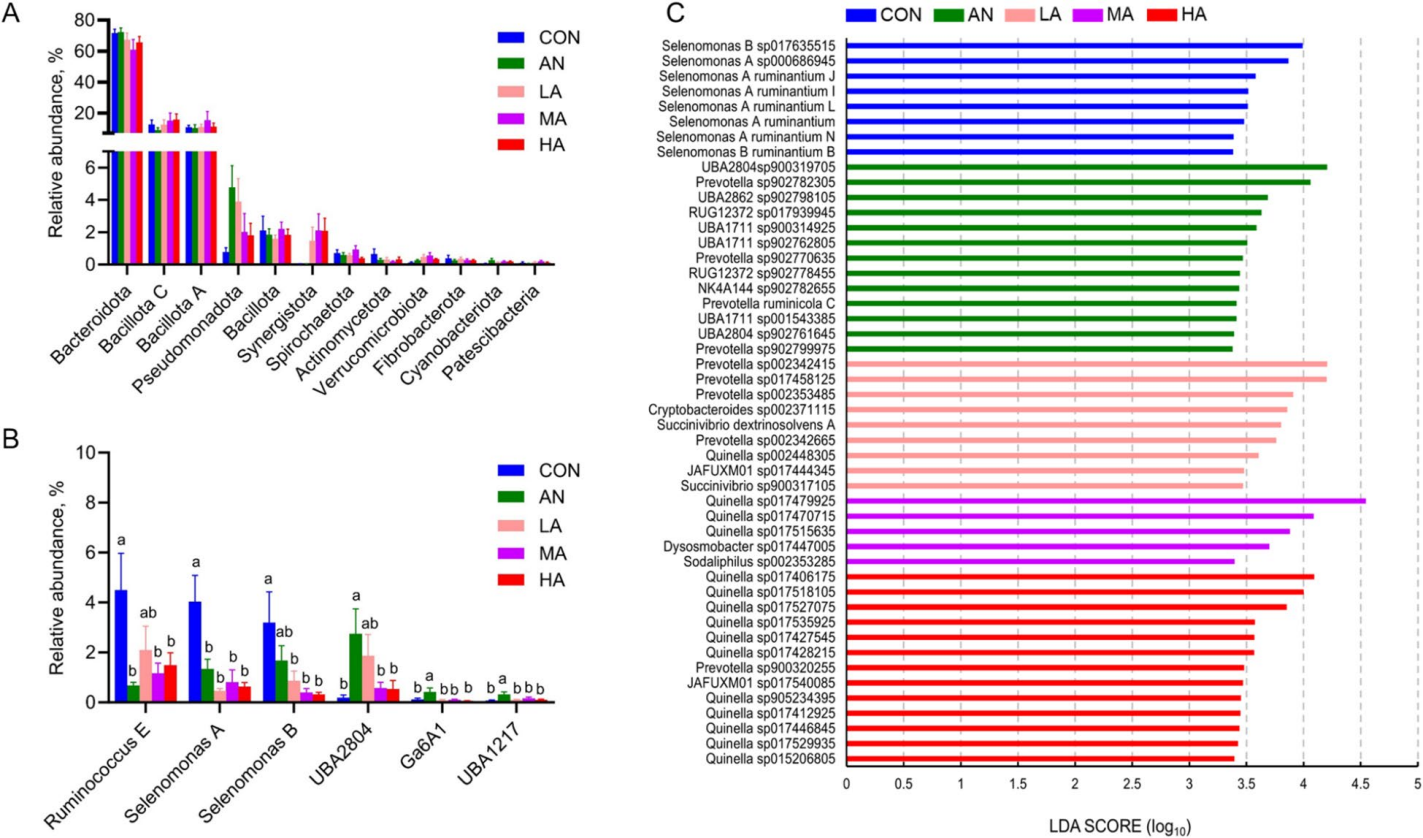
The effects of dietary treatments on rumen bacteria at the phylum (**A**), genus (**B**) and species (**C**) levels. CON = a control diet; AN = a control diet + 5,000 mg/kg chlortetracycline premix; LA = a control diet + 250 mg/kg *A*. *niger* cultures; MA = a control diet + 500 mg/kg *A*. *niger* cultures; HA = a control diet + 1,000 mg/kg *A*. *niger* cultures. ^a,b^Different superscripts indicate statistically significant differences (*P* < 0.05)

At the phylum level, rumen bacteria were mainly composed of Bacteroidota, Bacillota C, Bacillota A, Pseudomonadota, and Bacillota (Fig. 2A). For rumen microbial phyla exceeding 0.1% in relative abundance, the dietary groups indicated no marked differences (*P* > 0.05). Compared with the CON group, the ruminal abundances of *Ruminococcus E*, *Selenomonas A*, and *Selenomonas B* at the genus level were markedly lower (*P* < 0.05) in the MA and HA groups, while the abundance of *UBA*2804, *Ga*6*A*1 and *UBA*1217 in the AN group was higher (*P* < 0.05) than those in the CON, MA, and HA groups (Fig. 2B).

The influence of the dietary treatments on the rumen bacteria community (species level) is depicted in Fig. 2C. LEfSe analysis showed that a total of 48 strains were significantly (*P* < 0.05) affected by dietary treatments. Eight strains, such as *Selenomonas A ruminantium* and *Selenomonas B* sp017635515, were significantly enriched (*P* < 0.05) in the CON group. A total of 13 strains, including *Prevotella* sp902799975 and *Prevotella* sp902770635, exhibited significant enrichment in the AN group (*P* < 0.05). Nine strains, such as *Succinivibrio* sp900317105 and *Prevotella* sp002353485 showed marked enrichment (*P* < 0.05) in the LA group. *Quinella* sp017515635, *Sodaliphilus* sp002353285, and three other strains were significantly enriched in fattening sheep in the MA group (*P* < 0.05). Similarly, 13 strains, including *Quinella* sp015206805 and *Prevotella* sp900320255, showed marked enrichment in the HA group (*P* < 0.05).

### Composition of the rumen archaea

**Fig. 3:**
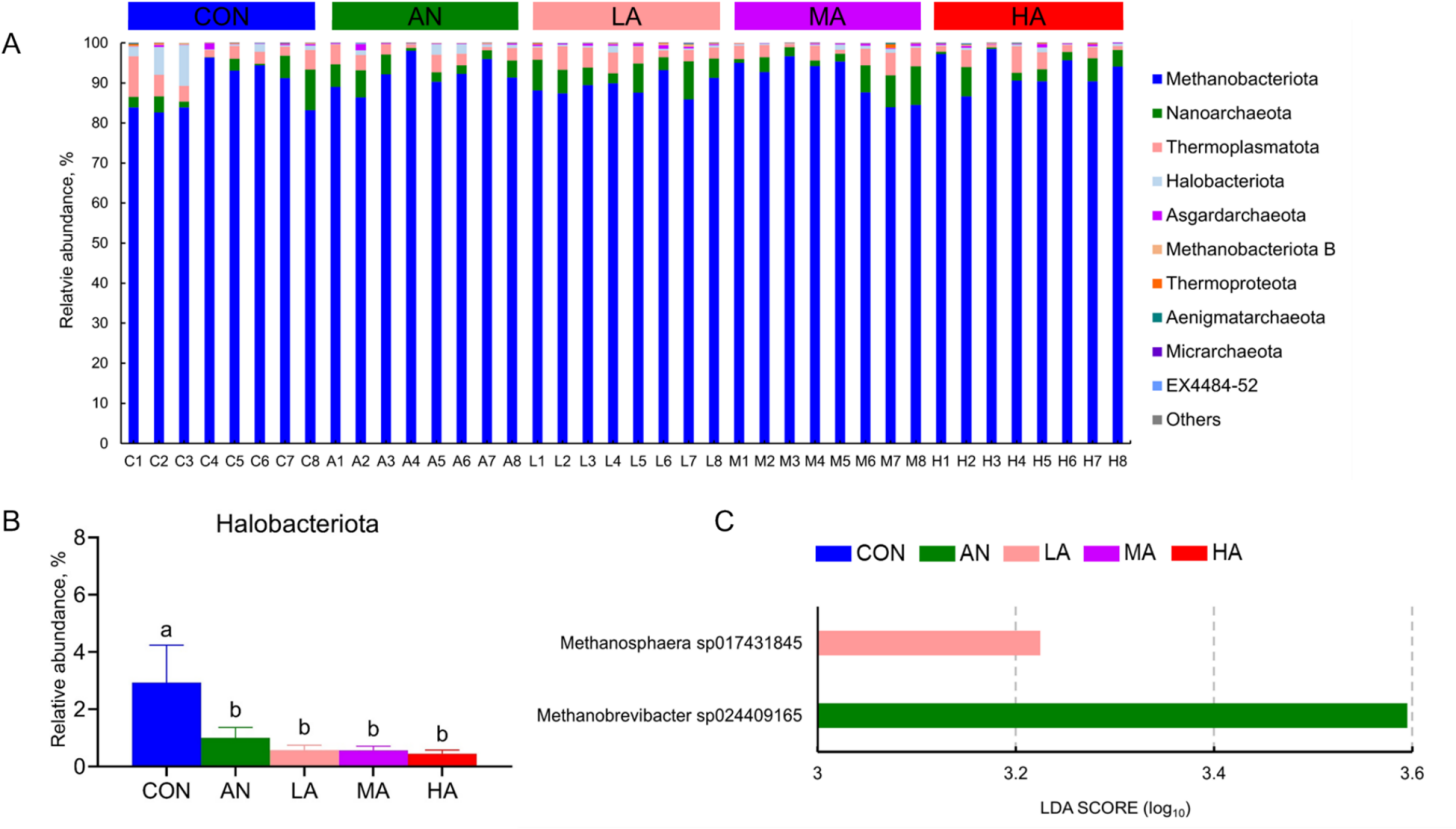
The effects of dietary treatments on rumen archaea at the phylum (**A** and **B**), and species (**C**) levels. CON = a control diet; AN = a control diet + 5,000 mg/kg chlortetracycline premix; LA = a control diet + 250 mg/kg *A*. *niger* cultures; MA = a control diet + 500 mg/kg *A*. *niger* cultures; HA = a control diet + 1,000 mg/kg *A*. *niger* cultures. ^a,b^Different superscripts indicate statistically significant differences (*P* < 0.05)

At the phylum level, rumen archaea of fattening sheep were mainly composed of Methanobacteriota, Nanoarchaeota, Thermoplasmatota, Halobacteriota, and Asgardarchaeota (Fig. 3A). Statistical analysis of the archaeal phyla with relative abundance greater than 0.1% revealed that the abundance of Halobacteriota in the rumen of fattening sheep in the AN and *A. niger* culture groups was significantly lower than in the CON group (*P* < 0.05, Fig. 3B). No significant differences (*P* > 0.05) were detected among the ration treatments for the other archaeal phyla. At the genus level, no significant effects (*P* > 0.05) of dietary treatments on archaea were observed. At the species level, LEfSe analysis showed that *Methanosphaera* sp017431845 was markedly enriched (*P* < 0.05) in the LA group and *Methanobrevibacter* sp024409165 was enriched (*P* < 0.05) in the AN group (Fig. 3C). No significantly enriched (*P* > 0.05) archaea strains were found in the rumen of fattening sheep in the CON, MA, and HA groups.

### Composition of rumen eukaryotes

**Fig. 4:**
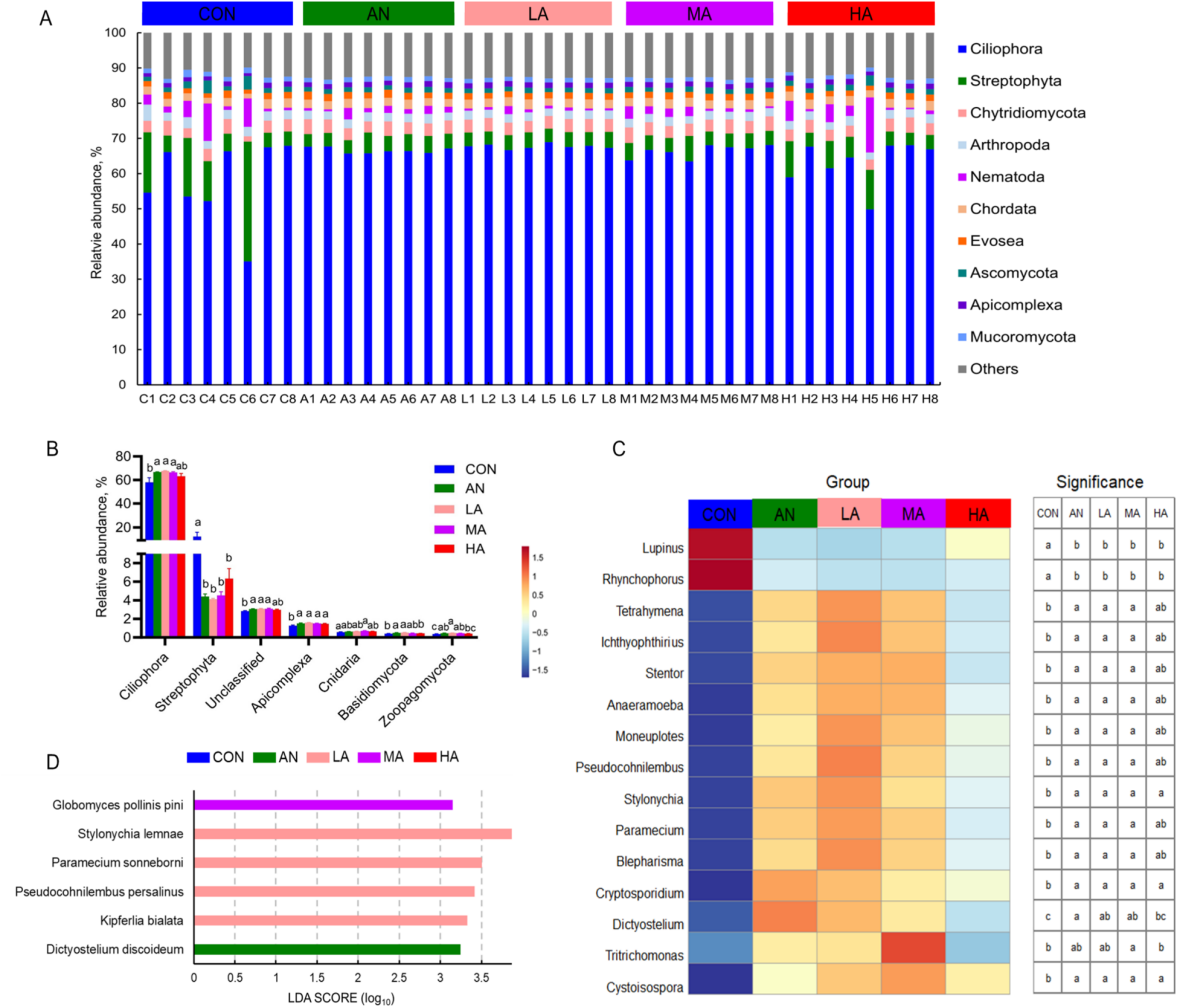
The effects of dietary treatments on rumen eukaryota at the phylum (**A **and** B**), genus (**C**) and species (**D**) levels. CON = a control diet; AN = a control diet + 5,000 mg/kg chlortetracycline premix; LA = a control diet + 250 mg/kg *A*. *niger* cultures; MA = a control diet + 500 mg/kg *A*. *niger* cultures; HA = a control diet + 1,000 mg/kg *A*. *niger* cultures. ^a–c^Different superscripts indicate statistically significant differences (*P* < 0.05)

At the phylum level, eukaryotes predominantly consisted of Ciliophora, Streptophyta, Chytridiomycota, Arthropoda, and Nematoda (Fig. 4A). Statistical analysis of the eukaryotic phyla with relative abundance greater than 0.1% revealed (Fig. 4B) that the abundance of Ciliophora was significantly higher in the AN, LA, and MA groups than in the CON group (*P* < 0.05). Compared with the CON group, the ruminal abundance of Streptophyta was significantly lower (*P* < 0.05), while that of Apicomplexa was higher (*P* < 0.05) in the AN and *A. niger* culture groups. The abundance of *Stylonychia*, *Cryptosporidium*, and *Cystoisospora* was significantly higher (*P* < 0.05) at the genus level, while the abundance of *Lupinus* and *Rhynchophorus* was significantly lower (*P* < 0.05) in the rumen of fattening sheep in the AN and *A. niger* culture groups as compared with the CON group (Fig. 4C). The abundance of *Tetrahymena*, *Ichthyophthirius*, *Stentor*, *Anaeramoeba*, *Moneuplotes*, *Pseudocohnilembus*, *Paramecium*, and *Blepharisma* was increased (*P* < 0.05) in the AN, LA, and MA groups compared with the CON group. At the species level (Fig. 4D), *Dictyostelium discoideum* showed significant enrichment (*P* < 0.05) in the AN group. In addition, *Kipferlia bialata, Pseudocohnilembus persalinus*, *Paramecium sonneborni*, and *Stylonychia lemnae* were enriched in the LA group (*P* < 0.05), while *Globomyces pollinis-pini* was enriched in the rumen of fattening sheep in the MA group (*P* < 0.05).

### Changes in carbohydrate active enzyme (CAZyme) profiles of rumen microbiota

**Fig. 5:**
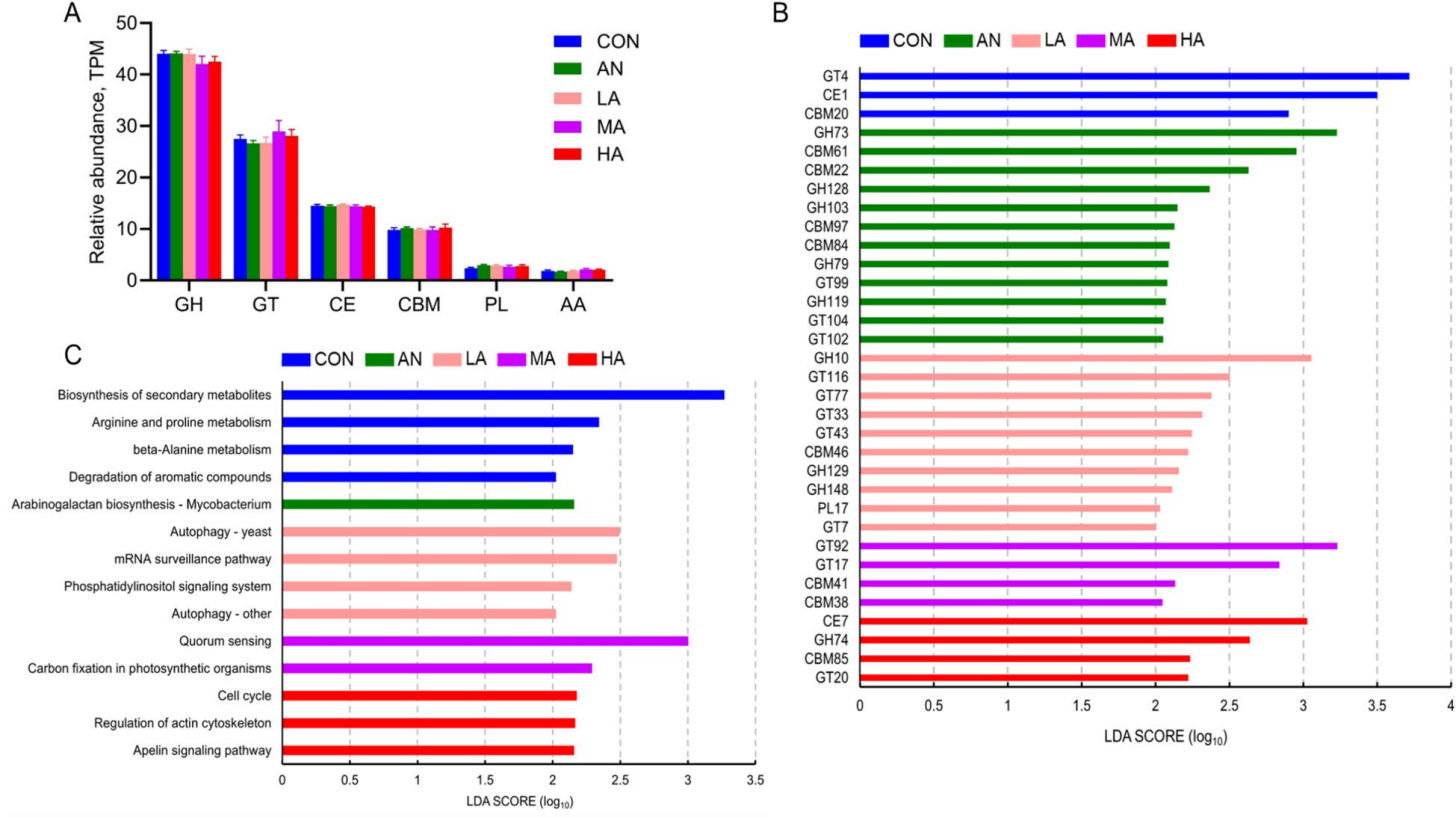
The effects of dietary treatments on rumen microbial functions. CAZymes at the class (**A**) and family (**B**) levels; Kyoto Encyclopedia of Genes and Genomes (KEGG) level 3 (**C**). CON = a control diet; AN = a control diet + 5,000 mg/kg chlortetracycline premix; LA = a control diet + 250 mg/kg *A*. *niger* cultures; MA = a control diet + 500 mg/kg *A*. *niger* cultures; HA = a control diet + 1,000 mg/kg *A*. *niger* cultures

At the class level, our results showed that the rumen microbial CAZymes were mainly composed of GH (average 42.61%), GT (average 26.79%), CE (average 14.18%), CBM (average 9.79%), and PL (average 2.70%) (Fig. S1C). However, statistical analysis revealed that different treatments did not affect the abundance of rumen microbial CAZymes in fattening sheep at the class level, as shown in Fig. 5A (*P* > 0.05).

PCoA coupled with PERMANOVA revealed no significant differences in CAZymes among the groups (*P* = 0.324, Fig. S1A). Furthermore, analysis at the family level showed that the rumen microbial CAZymes were predominated by GT2 (average 11.4%), CE1 (average 5.66%), GT4 (average 5.27%), GH13 (average 3.95%), and GH2 (average 3.32%) (Fig. S1D). As indicated by the statistical analysis (Fig. 5B), GT4, CE1, and CBM20 were enriched (*P* < 0.05) in the CON group. GH73, CBM61, CBM22, GH128, GH103, CBM97, CBM84, GH79, GT99, GH119, GT104, and GT102 revealed significant enrichment (*P* < 0.05) in the AN. The LA group showed significant enrichment mainly in GH10, GT116, GT77, GT33, GT43, CBM46, GH129, GH148, PL17, and GT7 (*P* < 0.05). GT92, GT17, CBM41, and CBM38 showed enrichment prominently in the MA group (*P* < 0.05). CE7, GH74, CBM85, and GT20 were enriched in the rumen of sheep in the HA group (*P* < 0.05).

### Alterations in KEGG pathways of rumen microbiota

At KEGG level 2 (Fig. S1E), rumen microbial categories mainly included global and overview maps (average 28.82%), carbohydrate metabolism (average 10.08%), amino acid metabolism (average 7.53%), metabolism of cofactors and vitamins (average 5.96%), and translation (average 5.22%). Moreover, PCoA and PERMANOVA revealed no significant variation in KEGG level 3 functional profiles among the groups (*P* = 0.695, Fig. S1B). At KEGG level 3 (Fig. S1F), rumen microbial categories mainly included metabolic pathways (average 18.53%), biosynthesis of secondary metabolites (average 8.43%), biosynthesis of antibiotics (average 5.47%), microbial metabolism in diverse environments (average 3.86%), and biosynthesis of amino acids (average 3.70%). Among them, degradation of aromatic compounds, arginine and proline metabolism, beta-alanine metabolism, and biosynthesis of secondary metabolites showed significant enrichment in the CON group (*P* < 0.05). Arabinogalactan biosynthesis–*Mycobacterium* was enriched in the rumen microbiota of fattening sheep in the AN group (*P* < 0.05). Autophagy–other, the phosphatidylinositol signaling system, the mRNA surveillance pathway, and autophagy–yeast exhibited significant enrichment in the LA group (*P* < 0.05). Carbon fixation in photosynthetic organisms and quorum sensing (QS) was prominently enriched (*P* < 0.05) in rumen microbiota of fattening sheep in the MA group. In contrast, the apelin signaling pathway, regulation of actin cytoskeleton, and the cell cycle pathway displayed significant enrichment in the HA group (*P* < 0.05, Fig. 5C).

### Alterations in microbial ARGs in the rumen

**Fig. 6:**
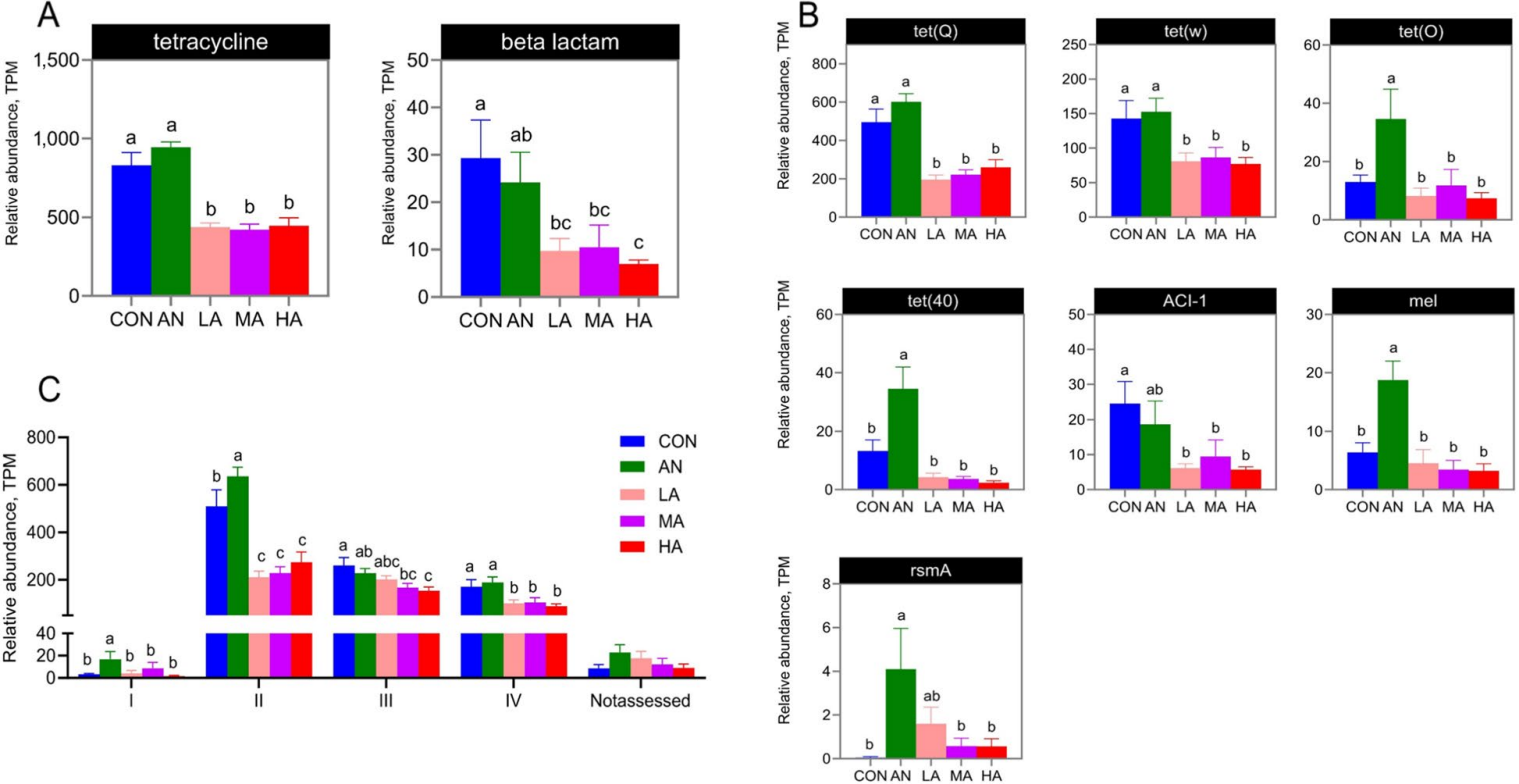
The effects of dietary treatments on rumen ARGs. **A** Changes in ARGs at the type level. **B** Changes in ARGs at the subtype level. **C** Changes in ARG ranks. CON = a control diet; AN = a control diet + 5,000 mg/kg chlortetracycline premix; LA = a control diet + 250 mg/kg *A*. *niger* cultures; MA = a control diet + 500 mg/kg *A*. *niger* cultures; HA = a control diet + 1,000 mg/kg *A*. *niger* cultures. ^a–c^Different superscripts indicate statistically significant differences (*P* < 0.05)

Analysis at the type level demonstrated that the *A. niger* culture groups harbored a significantly lower abundance (*P* < 0.05) of tetracycline ARGs in the rumen compared with the CON and AN groups (Fig. 6A and Fig. S2A), while no significant differences were observed between the rest of the groups (*P* > 0.05). A significantly lower β-lactam abundance was found in the HA group compared with both the CON and AN groups (*P* < 0.05). In contrast, the *A*. *niger* culture groups showed no significant differences (*P* > 0.05).

**Fig. 7:**
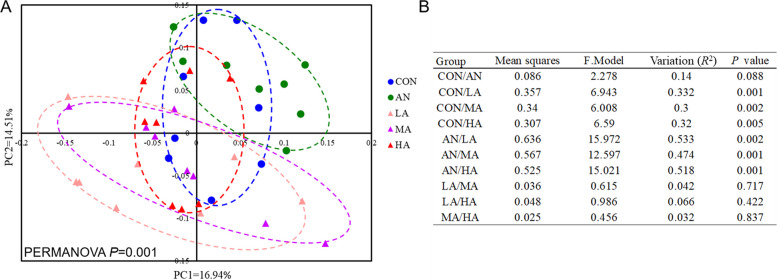
PCoA (**A**) and PERMANOVA (**B**) of ARGs. CON = a control diet; AN = a control diet + 5,000 mg/kg chlortetracycline premix; LA = a control diet + 250 mg/kg *A*. *niger* cultures; MA = a control diet + 500 mg/kg *A*. *niger* cultures; HA = a control diet + 1,000 mg/kg *A*. *niger* cultures

PCoA combined with PERMANOVA analysis indicated a trend towards a difference between the AN and CON groups (*P* = 0.088), whereas the *A. niger* culture groups were statistically significantly different from the CON and AN groups (*P* < 0.05, Fig. 7A and B). In addition, analysis at the subtype level showed that seven ARGs were significantly altered by the different ration treatments (*P* < 0.05). The abundance of rumen microbial *tet*(Q) and *tet*(W) in three *A. niger* culture groups of fattening sheep was lower than that of the CON and AN groups (*P* < 0.05), whereas the remaining groups did not differ significantly from one another (*P* > 0.05). The abundance of rumen microbial *tet*(O), *tet*(40), and *mel* in the AN group of fattening sheep was higher than that of the CON and *A. niger* culture groups (*P* < 0.05). The *A. niger* culture groups exhibited a significantly reduced abundance of the rumen microbiota *ACI-1* compared with the CON group (*P* < 0.05). A significantly lower abundance of rumen microbiota *rsmA* was observed in the MA, HA, and CON groups compared with the AN group (*P* < 0.05, Fig. 6B and Fig. S2B).

**Fig. 8:**
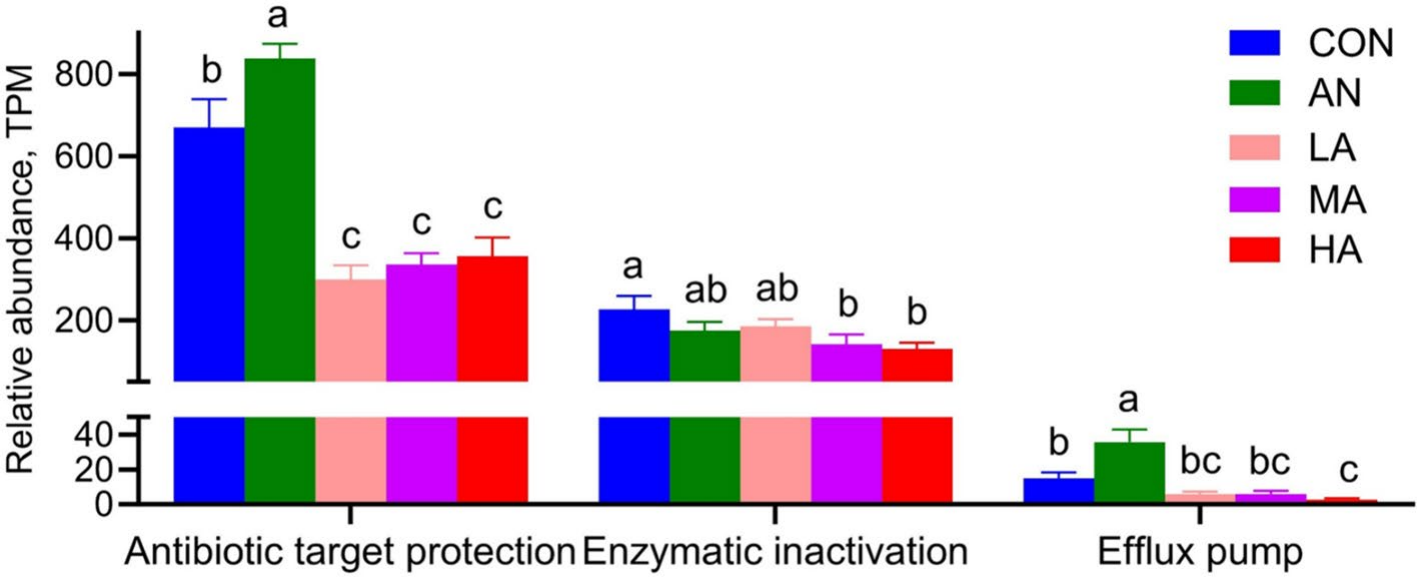
The effects of dietary treatments on the resistance gene mechanisms in the rumen of fattening sheep. CON = a control diet; AN = a control diet + 5,000 mg/kg chlortetracycline premix; LA = a control diet + 250 mg/kg *A*. *niger* cultures; MA = a control diet + 500 mg/kg *A*. *niger* cultures; HA = a control diet + 1,000 mg/kg *A*. *niger* cultures. ^a–c^Different superscripts indicate statistically significant differences (*P* < 0.05)

Furthermore, statistical analysis of rumen microbiota ARGs categorized by hierarchy revealed (Fig. 6C) that the abundance of Rank Ⅰ ARGs in the AN group was higher (*P* < 0.05) than that in the CON and *A. niger* culture groups. The AN group exhibited the highest abundance of Rank Ⅱ ARGs (*P* < 0.05), with both the AN and CON groups showing significantly greater (*P* < 0.05) abundance compared with the *A. niger* culture groups. Compared with the CON group, the abundance of Rank Ⅲ ARGs was significantly lower (*P* < 0.05) in the HA group, while the abundance of Rank Ⅳ ARGs in the *A. niger* culture groups was lower (*P* < 0.05) than that in both the CON and AN groups. No significant differences (*P* > 0.05) were observed among the other groups. Furthermore, results of the resistance gene mechanism in the rumen of fattening sheep (Fig. 8) revealed that, compared with the CON group, the activity of antibiotic target protection differed significantly (*P* < 0.05) in the AN group and the *A. niger* culture groups. Compared with the CON group, enzymatic inactivation activity was significantly lower in the MA and HA groups (*P* < 0.05). In addition, compared with the CON group, the activity of efflux pump mechanisms showed significant differences in the AN and HA groups (*P* < 0.05).

### Functional contributions of ARGs in rumen microbiota

In the present study, the results demonstrated that the abundance of tetracycline and β-lactam ARGs was significantly affected by dietary treatments. Therefore, we conducted a functional contribution analysis of these two types of ARGs. At the phylum level (Fig. S3A), functional contribution analysis of tetracycline ARGs showed that the major contributors were Pseudomonadota, Bacteroidota, and Bacillota A. Minor contributions were also detected from phyla such as Bacillota. *Tolumonas*, *Prevotella*, and *Eubacterium T* were identified as key contributors at the genus level (Fig. S3B). Other genera, including *Agathobacter*, *Peptacetobacter*, and *RUG*11690, also contributed, albeit to a smaller degree. Major contributors included *Tolumonas* sp008015085, *Eubacterium T pyruvativorans*, *Prevotella* sp900314755, and *Prevotella* sp002354095 at the species level (Fig. S3C). In contrast, species such as *Agathobacter* sp900549895, *Peptacetobacter hominis*, and *RUG*14300 sp024400635 demonstrated relatively low contributions.

An analysis of the functional contributions to β-lactam ARGs indicated that Bacillota C and Bacillota A were the dominant phyla (Fig. S3D). Bacteroidota also made a contribution, albeit minor. At the genus level, high-level contributors included *Succiniclasticum*, *RUG*14300, and *Oliverpabstia* (Fig. S3E). Key species contributing to β-lactam resistance were *Succiniclasticum* sp017405945, *RUG*14300 sp945482995, and *Oliverpabstia* sp022764165 (Fig. S3F). Several species, including *Sphingobacterium stercorigallinarum*, played a role as minor constituents.

### Rumen metabolomics

**Fig. 9:**
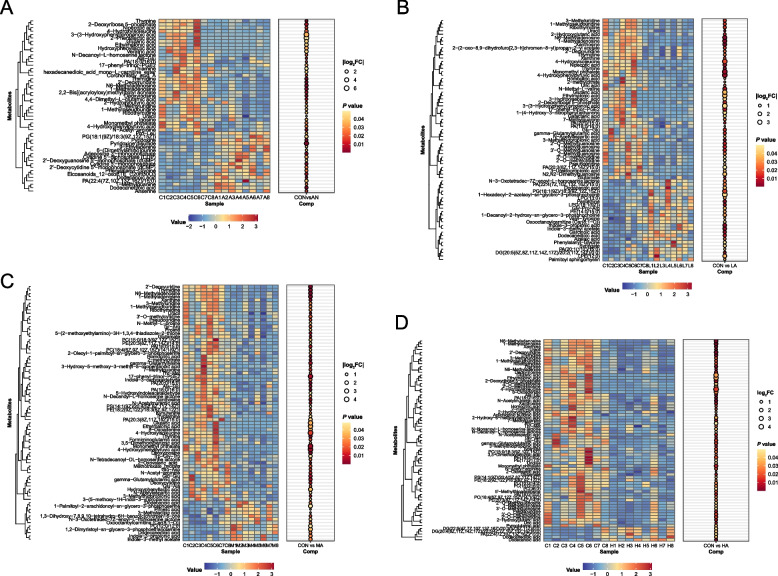
The effects of dietary treatments on rumen metabolites of fattening sheep. **A** The differential metabolites between the CON and AN groups. **B** The differential metabolites between the CON and LA groups. **C** The differential metabolites between the CON and MA groups. **D** The differential metabolites between the CON and HA groups. CON = a control diet; AN = a control diet + 5,000 mg/kg chlortetracycline premix; LA = a control diet + 250 mg/kg *A*. *niger* cultures; MA = a control diet + 500 mg/kg *A*. *niger* cultures; HA = a control diet + 1,000 mg/kg *A*. *niger* cultures

PLS-DA analysis revealed significant differences between the CON group and the AN, LA, MA, and HA groups (Fig. S4A). OPLS-DA analysis indicated that the CON group differed significantly from the other four groups, and that the AN group showed significant differences compared with the MA and HA groups (Fig. S4B). Furthermore, compared with the CON group, the rumen of the AN group fattening sheep exhibited significantly increased (*P* < 0.05) levels of compounds such as eicosanoids 12-oxoETE C20H30O3, pyridoxine disulfide, and pyridoxine, while levels of compounds such as N-decanoyl-L-homoserine lactone and thiamine were decreased (*P* < 0.05, Fig. 9A). In the rumen of the LA group fattening sheep, levels of indole-3-methyl acetate, indole-3-propionic acid, pantethine, garcinoic acid, and N-3-oxotetradec-7Z-enoyl-L-homoserine lactone were significantly higher than in the CON group (*P* < 0.05, Fig. 9B). Compared with the CON group, the MA group fattening sheep rumen showed significantly elevated (*P* < 0.05) levels of compounds such as indole-3-methyl acetate and indole-3-propionic acid, while levels of N-decanoyl-L-homoserine lactone, N-tetradecanoyl-DL-homoserine lactone, 5-(2-methoxyethylamino)-3H-1,3,4-thiadiazole-2-thione, tryptamine, and indole-3-carboxaldehyde were reduced (*P* < 0.05, Fig. 9C). The rumen of the HA group fattening sheep had significantly lower (*P* < 0.05) levels of compounds such as N-decanoyl-L-homoserine lactone, N-nonanoyl-L-homoserine lactone, and tyramine compared with the CON group (Fig. 9D).

### Alteration of microbiota–metabolite networks

**Fig. 10:**
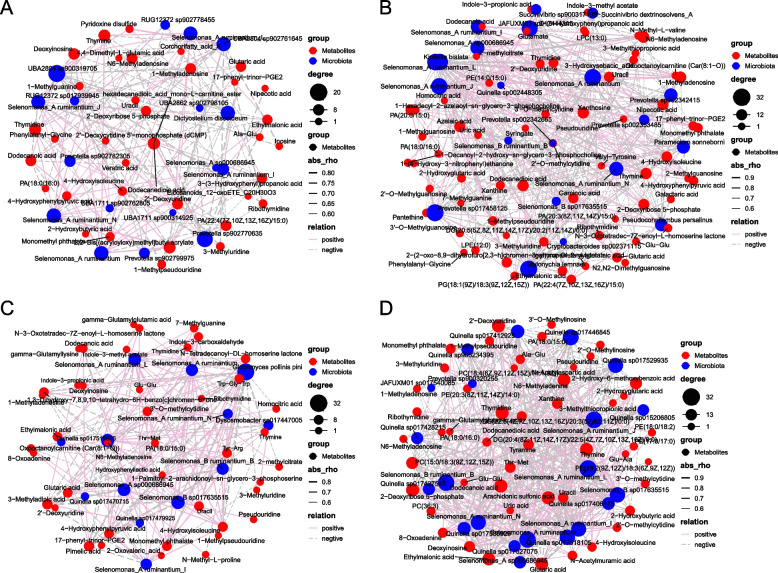
The effects of dietary treatments on association networks between microbiota and metabolites in the rumen of fattening sheep. **A** The association network between the CON and AN groups. **B** The association network between the CON and LA groups. **C** The association network between the CON and MA groups. **D** The association network between the CON and HA groups. CON = a control diet; AN = a control diet + 5,000 mg/kg chlortetracycline premix; LA = a control diet + 250 mg/kg *A*. *niger* cultures; MA = a control diet + 500 mg/kg *A*. *niger* cultures; HA = a control diet + 1,000 mg/kg *A*. *niger* cultures

Compared with the CON group, the association networks between rumen microbiota and metabolites exhibited distinct patterns in each treatment group (Fig. 10). The network in the AN group (Fig. 10A) consisted of 53 nodes and 173 edges, primarily featuring close connections between microbiota such as *UBA*2804 spp., *Prevotella* spp., and *Selenomonas A ruminantium* and metabolites including 2'-deoxycytidine 5'-monophosphate (dCMP). The LA group (Fig. 10B) displayed a more complex network with 86 nodes and 395 edges, in which *Prevotella* spp., *JAFUXM*01 spp., and *Selenomonas A ruminantium* were strongly associated with metabolites, such as indole-3-propionic acid. In the MA group (Fig. 10C), the network comprised 56 nodes and 194 edges, highlighting enhanced associations between microbiota such as *Globomyces pollinis-pini*, *Selenomonas A ruminantium* and metabolites including indole-3-propionic acid and thymine. The HA group (Fig. 10D) exhibited the most intricate network, with 71 nodes and 455 edges, where microbiota, associated with *Quinella* spp. and *Selenomonas* spp., showed strong correlations with metabolites such as tyramine and thymine.

## Discussion

### Effects of *A*.* niger* cultures on the growth performance of fattening sheep

Since China prohibited antibiotic use in animal feed in 2020, researchers have sought safer and more efficient alternatives to support animal health and optimize production performance. As a functional fungus with probiotic properties, *A. niger* metabolizes to produce multifunctional enzymes and bioactive substances that demonstrate significant regulatory effects on animal health and growth performance [21]. Hence, *A. niger* preparations or cultures have attracted much attention from researchers in recent years. Our findings suggest that, compared with the CON group, the AN and HA groups significantly increased the ADG of fattening sheep. Pittaluga and Pelling [22] found that supplementing the basal diet of beef cattle with 0.02% of a fungal feed additive rich in *A*. *niger* increased ADG after a 122-day trial period, with a significant cubic response. Similarly, Wang et al. [23]. demonstrated that the compound probiotics with *A. oryzae* significantly boosted ADG in Hu sheep. Overall, the above results revealed that *A. niger* cultures had a certain promoting effect on the growth performance of sheep and exhibited promising potential as an antibiotic alternative in ruminants.

### Effects of *A*.* niger* cultures on blood biochemical, antioxidant, and immunological indices in fattening sheep

Subsequently, we systematically evaluated the effects of *A. niger* cultures on the host metabolism of fattening sheep by measuring blood biochemical, antioxidant, and immunological indices. In the present study, our results indicated that *A. niger* cultures increased the concentrations of plasma TP and GLB in sheep compared with the AN group. TP is a key biochemical indicator for assessing the physiological status of the animal body and disease diagnosis [24]. In addition, GLB, an important component of TP, is mainly involved in physiological processes, such as immune defense and nutrient transport [25]. Similar to our results, one study proved that dietary supplementation with *A. oryzae* extract resulted in a significant increase in the plasma TP level, along with an upward trend in the GLB level of cows [26]. Furthermore, another study showed that after intramuscular injection of oxytetracycline (a tetracycline family, the same as chlortetracycline), the TP level in goat serum was significantly reduced [27]. Thus, the above results indicated that *A. niger* cultures may be more beneficial for enhancing the immune system of fattening sheep than antibiotic supplementation.

In addition, our results on antioxidant indices indicated that the MA and HA groups had a significantly higher plasma SOD level than the CON group. Among the antioxidant enzyme systems, an increase in SOD activity can effectively eliminate ROS from the animal, resulting in an antioxidant effect [28]. Consistent with our result, a previous study found that dietary supplementation with *A*. *niger* and its compound preparations significantly increased the SOD activity in bulls [29]. Thus, the above results indicated that *A. niger* cultures possessed a certain antioxidant capacity and had positive effects on sheep health.

### Effects of *A*.* niger* cultures on the composition and diversity of rumen microbiota in fattening sheep

The rumen microbial community is primarily composed of three major groups—bacteria, archaea, and eukaryotes—which play critical roles in maintaining rumen microecological stability and facilitating nutrient metabolism [30]. The concentrate portion of ruminant diets is predominantly starch based. Hespell et al. [31] found *Succinivibrio* to be a key bacterial group involved in starch degradation in the rumen, with acetate and succinic acid being the principal fermentation end products. Therefore, the enrichment of *Succinivibrio dextrinosolvens A* and *Succinivibrio* sp900317105 in the LA group suggested that *A. niger* could enhance the starch-degrading function in the rumen. In addition, *Prevotella*, widely present in the rumen, is another important microbiota involved in starch degradation and utilization [32]. Meanwhile, *Prevotella* harbors highly active hemicellulose-degrading strains and plays a significant role in hemicellulose degradation [33]. Moreover, previous studies reported that *Prevotella* possessed a broad repertoire of genes coding for enzymes/proteins responsible for the pretreatment of cellulose, starch, and lignocellulose [34]. In the present study, several *Prevotella* species (such as *Prevotella* sp902799975, *Prevotella* sp002353485, and *Prevotella* sp900320255) were significantly enriched in the rumen of the AN, LA, and HA groups. These results indicated that *A. niger* cultures may enhance the degradation capacity of starch, hemicellulose, and cellulose by promoting the colonization of these specific *Prevotella* spp. in the rumen of sheep. Moreover, *Quinella*, a signature hydrogenotrophic bacterium in the rumen, has been reported to utilize sugars to produce propionate through the fermentation pathway [35]. The studies confirmed that *Aspergillus* spp. have been shown to promote succinic acid production by boosting the degradation of fiber and providing *Quinella* with metabolic substrates (e.g., soluble sugars) [36, 37]. In a previous study, *Quinella* was reported to be closely associated with low methane production in sheep [37]. Hence, in the present study, the significant enrichment of several *Quinella* species (such as *Quinella* sp017515635 and *Quinella* sp015206805) in the MA and HA groups suggested that *A. niger* cultures may enhance rumen fiber degradation capacity.

Regarding archaea, we found that *Methanobrevibacter* sp024409165 and *Methanosphaera* sp017431845 were significantly enriched in the AN and LA groups, respectively. Methane production is closely associated with methanogenic archaea and the availability of hydrogen [38]. As mentioned earlier, the hydrogen-utilizing bacteria *Quinella* spp. were enriched in the MA and HA groups, but we did not find any methanogens enriched in these two groups. Therefore, we thought that the effect of *A. niger* cultures on methane production was uncertain and that its underlying mechanism required further investigation. Within the rumen eukaryotic community, a marked increase in the protozoan population was indicated, particularly in the relative abundance of phylum Ciliophora and its several genera (such as *Tetrahymena*, *Ichthyophthirius*, *Stentor*), following dietary supplementation with AN or *A. niger* cultures. Previous studies have indicated that fungal additives may serve as potent prebiotics to stimulate rumen protozoal growth, likely due to the bioactive compounds in fungal metabolites, including soluble factors and cell membrane-associated mannan and β-glucan [39, 40]. On the other hand, we thought that the higher pH in the AN and HA groups also contributed to the proliferation of rumen protozoa. The results of studies on high-concentrate diets suggested that long-term low pH was probably more detrimental to the survival of rumen protozoa populations than other factors [41, 42]. Overall, in the present study, dietary supplementation with *A. niger* cultures led to an increase in the protozoa population in the rumen, either directly or indirectly. Previous studies indicated that protozoa reduced the degradation rate of starch by engulfing starch granules, thereby increasing the amount of starch in the small intestine and helping maintain ruminal ecological balance in ruminants fed high-grain diets [43]. Hence, an increase in the protozoal population observed in the rumen of fattening sheep from the AN or *A. niger* culture groups may regulate starch degradation and contribute to rumen environmental stability under high-concentrate diets in the present study.

### Effects of *A*.* niger* cultures on changes in the characteristics of CAZymes in fattening sheep

CAZymes represent the core enzyme systems utilized by the rumen microbiota to degrade carbohydrates (such as starch, cellulose and hemicellulose), primarily including the following six categories: GHs, PLs, CEs, AAs, CBMs, and GTs [44]. Among the GH family, GH10 maintains stability across a wide pH range and activities toward xylans and cellulosic substrates [45]. GH148 is categorized as an endoglucanase family and is closely associated with fiber degradation [46]. Interestingly, the primary function of GH74 is to degrade xyloglucan, a major component of hemicellulose. By cleaving the linkages within xyloglucan, GH74 disrupts the connections between xyloglucan and hemicellulose, thereby loosening the rigid structure of the plant cell wall and releasing oligosaccharides and monosaccharides [47, 48]. In the present study, GH10 and GH148 in the LA group, as well as GH74 in the HA group, were significantly enriched in the rumen of fattening sheep. Therefore, we considered that *A. niger* could promote the digestion of plant fibers in ruminants and provide sufficient energy and carbon sources for rumen microbiota. Within the CBM family, CBM20 is uniquely associated with GH13 α-amylases and GH15 glucoamylases as a starch-binding module, while CBM41 contains multiple repeated amylase domains similar to those in CBM20 [46]. In addition, GH119 has been confirmed to possess abundant α-amylase activity and to participate in the starch saccharification process [46]. In the present study, CBM20 in the CON group, CBM41 in the MA group, and GH119 in the AN group were all significantly enriched in the rumen of the fattening sheep. In the present study, all experimental diets consisted of high-concentrate rations; it was plausible that the differential dietary treatments resulted in the expression of distinct enzymatic profiles responsible for starch degradation. Overall, the above results demonstrated that *A. niger* cultures may promote the degradation of dietary fiber and starch in sheep rumen by modulating enzyme composition.

### Effects of *A*. *niger* cultures on the KEGG functions of rumen microbiota in fattening sheep

Next, to further understand the changes in rumen microbiota, we analyzed the KEGG functions of rumen microbiota. Our results showed that pathways including autophagy–other, autophagy–yeast, phosphatidylinositol signaling system, quorum sensing, and cell cycle were significantly enriched in groups supplemented with different doses of *A. niger* cultures. The autophagy pathway serves as a key mechanism for cellular homeostasis, degrading damaged organelles or misfolded proteins to recycle nutrients and sustain cell survival [49]. The phosphatidylinositol signaling system and apelin signaling pathway are critical signal transduction systems involved in regulating cell proliferation and differentiation, modulating energy metabolism, and apoptosis inhibition [50, 51]. Moreover, QS regulates microbial group behaviors through the secretion and perception of signaling molecules, reflecting the metabolic status and interactions of rumen microbiota [52]. Notably, microbiota possess the ability to adapt to nutrient availability by regulating cell cycle parameters, and this capability is closely linked to their survival and proliferation [53]. Therefore, the enrichment of the aforementioned KEGG pathways in the LA, MA, and HA groups indicated that *A. niger* cultures may enhance the adaptive and regulatory capabilities of sheep rumen microbiota. This adaptation may promote more efficient nutrient degradation and absorption while contributing to a balanced rumen environment.

### Effects of *A*.* niger* cultures on the ARGs of rumen microbiota in fattening sheep

ARGs are genetic segments present in bacterial genomes that confer the ability to resist antibiotic killing or inhibition, and these genes can be transmitted and infect humans. Within animal hosts (e.g., the intestines), the horizontal transfer of ARGs among diverse bacterial species is primarily mediated by mobile genetic elements, including plasmids, transposons and integrons [54, 55]. In the field of animal husbandry, decreasing AMR and eliminating antimicrobial residues throughout the food production chain constitute fundamental safeguards for ensuring food safety and human public health [56]. Our data on ARGs demonstrated a significant decrease in the abundance of both tetracycline and β-lactam resistance genes in the rumen microbiota of the *A. niger* culture groups at the type level. Simultaneously, *tet*(Q) and *tet*(W) were reduced in the rumen microbiota of the *A. niger* culture groups at the subtype level, while those of *tet*(O), *tet*(40), and *mel* were markedly elevated in the AN compared with other groups. Our results confirmed that the use of AN significantly increased the abundance of ARGs, while dietary supplementation with *A. niger* cultures markedly decreased the abundance of ARGs in the rumen microbiota of sheep.

Actually, only a small subset of ARGs are capable of transferring to other bacteria, especially human pathogens, and subsequently pose a threat to humans [57]. Rank I ARGs pose the greatest threat to human health. For this purpose, Zhang et al. [58] established a hazard ranking system, categorizing ARGs into three ranks based on their enrichment in human-related environments, mobility potential, and pathogenicity. Our results showed that the AN group consistently demonstrated higher abundances of both Rank I and Rank II ARGs compared to the other groups, and the abundances of Rank Ⅱ and Rank Ⅳ ARGs in the *A. niger* culture groups were lower than in the CON and AN groups. Hence, our results revealed that the use of antibiotics indeed increased the risk of spreading ARGs and even jeopardized human health, while dietary supplementation with *A. niger* reduced this risk.

### Effects of *A*.* niger* cultures on rumen metabolomics in fattening sheep

QS has been reported to be a mode of communication between bacteria, mainly through the secretion and detection of signaling molecules between bacterial populations, and the coordinated behavior that results from the establishment of population size [59]. *A. niger* metabolizes to produce β-glucan which is degraded to glucose, indirectly providing a carbon source for the bacteria and activating LuxI-type synthase, which may promote the synthesis of long-chain AHLs [e.g., N-3-oxotetradec-7Z-enoyl-L-homoserine lactone (3-oxo-C14:1-HSL)] [60, 61]. The level of 3-oxo-C14:1-HSL in the LA group was significantly higher. In the non-rumen environment, 3-oxo-C14:1-HSL was reported to inhibit bacterial biofilm formation, so we considered that a similar response may be present in the rumen, which would reduce the colonization ability of pathogenic bacteria (e.g., *E*. *coli*, *Salmonella*) and optimize the homeostasis of the rumen environment. Moreover, a prior study suggested that antibiotics may hinder the synthesis of AHLs by inhibiting the secretion of precursors (e.g., starch-degrading bacteria, proteolytic bacteria) and decreasing the fermentation products (e.g., fatty acids, S-adenosylmethanethionine) [62]. Simultaneously, *A. niger* is clearly identified as a known AHL-degrading fungus, mentioning that they may utilize enzymes such as lactonase and acyltransferase to cleave key chemical bonds (lactone ring or amide bond) of the AHL molecule [63]. In the present study, N-decanoyl-L-homoserine lactone (C10-HSL) was significantly reduced in the rumen of fattening sheep in the MA, HA, and AN groups. Another study found that reducing medium-chain AHLs (e.g., C10-HSL) could inhibit the QS signaling of pathogenic bacteria through quorum quenching, thereby significantly inhibiting the expression of α-toxin genes and biofilm formation in *Clostridium perfringens*, which ultimately stabilized the rumen microecology [64]. Hence, the reduced C10-HSL level in the rumen of the MA, HA, and AN groups indicated that antibiotics and *A. niger* may inhibit pathogen proliferation to some extent through the QS mechanism. In summary, our results showed that *A. niger* could help maintain rumen microbiota homeostasis, although the specific mechanisms have not yet been fully clarified.

Moreover, the alterations in thiamine and biogenic amines after *A. niger* cultures and antibiotics attracted our attention in the present study. Thiamine, a water-soluble micronutrient, is essential for a diverse range of physiological processes, including cell growth and development, rumen bacterial growth, rumen functions, cellular immunity, and inflammation [65]. However, Schwab et al. [66] found that feeding high-concentrate diets could reduce thiamine concentrations in the rumen of cows; insufficient levels may cause thiamine deficiency. Previous studies confirmed that thiamine could alleviate or mitigate high-concentrate feeding-induced rumen epithelial inflammation or damage by inhibiting the expression of TLR-NF-κB pathway-related genes or proteins [67]. In the present study, the thiamine concentration in the AN group was markedly reduced, but this reduction did not occur after *A. niger* culture supplementation. The above results showed that antibiotic supplementation did not alleviate the reduction of thiamine content caused by high-concentrate diets, whereas *A. niger* cultures supplementation did. Biogenic amines are mainly produced through the decarboxylation of amino acids by rumen microbiota, including tryptamine, tyramine, and histamine [68]. Biogenic amines are considered significant contributors to the epithelial damage and inflammatory responses induced by high-concentrate diets in the rumen, and the absorption of biogenic amines further triggers a series of systemic inflammatory reactions [69]. Hence, in this study, we found that tryptamine and tyramine concentrations were significantly decreased in the rumen of the MA and HA groups, suggesting that *A. niger* cultures may reduce damage to the rumen epithelium caused by high-concentrate diets by decreasing the biogenic amine content. Taken together, our findings indicate that *A. niger* cultures are beneficial for maintaining a healthy rumen environment under high-concentrate feeding conditions.

Interestingly, we observed that *A. niger* cultures had a certain impact on tryptophan metabolism, specifically manifested as an increase in indole-3-propionic acid and indole-3-methyl acetate contents in the LA and MA groups, and a decrease in 5-hydroxyindoleacetaldehyde content in the MA group. Indole-3-propionic acid (IPA), an indole derivative metabolized by gut microbiota from tryptophan, acts as an aryl hydrocarbon receptor (AhR) ligand, mediating regulatory T-cell differentiation and suppressing inflammatory responses [70]. In addition, a study on in vitro cell culture showed that IPA could regulate human intestinal barrier function and inflammation through the xenobiotic sensor pregnane X receptor [71]. Indole-3-acetic acid (3-IAA) has been reported to modulate immune homeostasis and reduce oxidative stress in the gut [72], and methyl indole-3-acetate is a methylated ester derivative formed from 3-IAA. Although there are limited studies on methyl indole-3-acetate at present, existing studies indicate that methyl indole-3-acetate has a protective effect against ulcerative colitis [73]. Hence, elevated ruminal IPA and methyl indole-3-acetate concentrations in the LA and MA groups of fattening sheep indicated that *A. niger* cultures may confer immunomodulatory effects and generate positive effects on rumen health. Moreover, 5-hydroxytryptamine, an important intermediate in tryptophan metabolism, is essential for maintaining normal intestinal function and suppressing inflammatory development [74]. The role of 5-hydroxytryptamine is terminated by catalytic metabolism into 5-hydroxyindoleacetaldehyde by the enzyme kynurenine 3-monooxygenase [75]. Hence, the decreased content of 5-hydroxyindoleacetaldehyde in the rumen of the MA group suggests that *A. niger* cultures may enhance the effects of 5-hydroxytryptamine, which is beneficial for rumen immunity and health.

In addition, we conducted microbiota–metabolite networks in each dietary treatment group. Our results demonstrated that, compared with the AN group, the association networks between rumen microbiota and metabolites exhibited distinct patterns in the *A. niger* culture groups. These results indicated that supplementation with *A. niger* cultures significantly enhanced the connectivity and interactions between rumen microbiota and metabolites, demonstrating the potential of *A. niger* cultures to modulate functional interactions within the rumen microecosystem.

### Study limitations

Although our study preliminarily and systematically investigated the effects of *A. niger* cultures on growth performance, rumen microbial composition, ARGs, and metabolic profiles in fattening sheep, the study still had the following limitations. First of all, we found a small number of plant- and insect-associated taxa in the metagenomic data, which may be related to the dietary characteristics of fattening sheep. This may be an inherent limitation of metagenomics, suggesting that we should integrate other methods such as microbial culturomics in the future to jointly verify microbiome results, and avoid bias in microbiological results caused by a single method. Second, as this was a preliminary study, the relatively small sample size may have introduced statistical bias and limited the ability to detect subtle but biologically significant changes in the microbial communities and ARG profiles. Additionally, as this investigation was conducted using a single breed of fattening sheep raised under specific management conditions, the generalizability of our findings to other breeds or production systems remains uncertain. Future large-sample studies involving other ruminant breeds and farms are necessary to validate and expand upon these preliminary findings.

## Conclusion

This study revealed that *A. niger* cultures effectively enhanced the growth performance and antioxidant capacity of fattening sheep, modulated the ruminal microbial community structure, markedly decreased the abundance of ARGs, and promoted the accumulation of beneficial metabolites in the rumen. Specifically, the *A. niger* cultures promoted the proliferation of key microbiota involved in starch and fiber degradation and enhanced the production of metabolites associated with immune regulation. Furthermore, the *A. niger* cultures significantly reduced the abundance of high-risk ARGs associated with tetracycline and β-lactam antibiotics, and the microbial consortia responsible for this reduction in ARGs were further identified. As a potential antibiotic alternative, *A. niger* cultures hold significant application value, offering the dual benefit of mitigating antibiotic resistance and helping maintain the stability of the ovine ruminal microbiota, thereby ultimately improving the overall production performance of fattening sheep. However, due to the limited sample size and single-breed design, these findings should be considered exploratory. Further research with larger sample sizes and involving different ruminant breeds is necessary to confirm the reproducibility and generalizability of the observed effects.

## Supplementary Information

Additional file 1: Table S1. Ingredients and chemical composition of the basal diets. 

Additional file 2: Fig. S1. Principal coordinates analysis (PCoA) and permutational multivariate analysis of variance (PERMANOVA) of carbohydrate active enzymes (CAZymes) (A) and Kyoto encyclopedia of genes and genomes (KEGG) level 3 (B), and the effects of dietary treatments on the relative abundance of rumen microbial CAZymes at the class (C) and family (D) levels, microbial categories at the KEGG level 2 (E), and KEGG level 3 (F). Fig. S2. Effects of dietary treatments on the relative abundance of rumen antibiotics resistance genes (ARGs) at the type level (A) and subtype level (B). Fig. S3. Effects of dietary treatments on functional contribution of tetracycline resistance genes in rumen at the phylum level (A), genus level (B) and species level (C), and on that of β-lactam resistance genes in rumen at the phylum level (D), genus level (E) and species level (F). Fig. S4. Effects of dietary treatments on differences in rumen microbial metabolites by partial least squares discriminant analysis (PLS-DA) (A) and orthogonal partial least squares discriminant analysis (OPLS-DA) (B). 

## Data Availability

Data will be made available on request.

## Abbreviations

ADFI: Average daily feed intake
ADG: Average daily gain
AhR: Aryl hydrocarbon receptor
ALB: Albumin
ALP: Alkaline phosphatase
ALT: Alanine aminotransferase
AMR: Antimicrobial resistance
*A*. * niger*: *Aspergillus niger*
AN: Antibiotic
*A*. * oryzae*: *Aspergillus oryzae*
ARGs: Antibiotic resistance genes
AST: Aspartate aminotransferase
BUN: Blood urea nitrogen
BW: Body weight
CAT: Catalase
CAZymes: Carbohydrate active enzymes
dCMP: 2'-Deoxycytidine 5'-monophosphate
F/G: Feed to gain ratio
FBW: Final body weight
GLB: Globulin
GLU: Glucose
GSH-Px: Glutathione peroxidase
HDL: High-density lipoprotein
IBW: Initial body weight
IgA: Immunoglobulin A
IgG: Immunoglobulin G
IgM: Immunoglobulin M
IPA: Indole-3-propionic acid
KEGG: Kyoto Encyclopedia of Genes and Genomes
LDH: Lactate dehydrogenase
LDL: Low-density lipoprotein
MCP: Microbial protein
MDA: Malondialdehyde
NH3-N: Ammonia nitrogen
OPLS-DA: Orthogonal partial least squares discriminant analysis
PERMANOVA: Permutational multivariate analysis of variance
PCoA: Principal coordinate analysis
PLS-DA: Partial least squares discriminant analysis
QS: Quorum sensing
SOD: Superoxide dismutase
T-AOC: Total antioxidant capacity
TC: Total Cholesterol
TG: Triglycerides
TP: Total protein
TVFA: Total volatile fatty acids
VFA: Volatile fatty acids
3-IAA: Indole-3-acetic acid
3-oxo-C14:1-HSL: N-3-Oxotetradec-7Z-enoyl-L-homoserine lactone

## References

[1] Caton JS, Crouse MS, Dahlen CR, Ward AK, Diniz WJS, Hammer CJ, et al. Micronutrient supply, developmental programming, and strategic supplementation in ruminant livestock. Animal. 2025;19(Suppl 2):101563. 10.1016/j.animal.2025.101563. 40579314 10.1016/j.animal.2025.101563

[2] Sun H, Wu YM, Wang YM, Wang C, Liu JX. Effects of addition of *Aspergillus oryzae* culture and 2‐hydroxyl-4-(methylthio) butanoic acid on milk performance and rumen fermentation of dairy cows. Anim Sci J. 2017;88(4):602–9. 10.1111/asj.12646. 27506446 10.1111/asj.12646

[3] Zhang JY, Jin W, Jiang Y, Xie F, Mao SY. Response of milk performance, rumen and hindgut microbiome to dietary supplementation with *Aspergillus oryzae* fermentation extracts in dairy cows. Curr Microbiol. 2022;79(4):113. 10.1007/s00284-022-02790-z. 35184209 10.1007/s00284-022-02790-z

[4] Podversich F, Tarnonsky F, Bollatti JM, Silva GM, Schulmeister TM, Martinez JJV, et al. Effects of *Aspergillus oryzae* prebiotic on animal performance, nutrients digestibility, and feeding behavior of backgrounding beef heifers fed with either a sorghum silage- or a byproducts-based diet. J Anim Sci. 2023;101:skac312. 10.1093/jas/skac312. 10.1093/jas/skac312 PMC9922046 36773039

[5] Yu RL, Liu J, Wang Y, Wang H, Zhang HW. *Aspergillus niger* as a secondary metabolite factory. Front Chem. 2021;9:701022. 10.3389/fchem.2021.701022. 34395379 10.3389/fchem.2021.701022 PMC8362661

[6] Yerby SE, Huntington J, Warren H, Jonsson NN. The effects of a product of the solid-state fermentation of *Aspergillus niger* on the *in vitro* rumen fermentation kinetics of rations fed to dairy cattle. Animal-Open Space. 2025;4:100098. 10.1016/j.anopes.2025.100098. 10.1017/S0022029925101313 40959966

[7] Kong FL, Lu N, Liu YF, Zhang S, Jiang HQ, Wang HM, et al. *Aspergillus oryzae* and *Aspergillus niger* co-cultivation extract affects *in vitro* degradation, fermentation characteristics, and bacterial composition in a diet-specific manner. Animals. 2021;11(5):1248. 10.3390/ani11051248. 33926015 10.3390/ani11051248 PMC8145302

[8] Zhuang YM, Guo W, Cui K, Tu Y, Diao QY, Zhang NF, et al. Altered microbiota, antimicrobial resistance genes, and functional enzyme profiles in the rumen of yak calves fed with milk replacer. Microbiol Spectr. 2024;12(1):e01314–23. 10.1128/spectrum.01314-23. 38014976 10.1128/spectrum.01314-23 PMC10871699

[9] Zhao YC, Li LX, Tan J, Zhao HY, Wang Y, Zhang A, et al. Metagenomic insights into the inhibitory effect of phytochemical supplementation on antibiotic resistance genes and virulence factors in the rumen of transition dairy cows. J Hazad Mater. 2025;490:137717. 10.1016/j.jhazmat.2025.137717. 10.1016/j.jhazmat.2025.137717 40020294

[10] Zaheer R, Lakin SM, Polo RO, Cook SR, Larney FJ, Morley PS, et al. Comparative diversity of microbiomes and resistomes in beef feedlots, downstream environments and urban sewage influent. BMC Microbiol. 2019;19:197. 10.1186/s12866-019-1548-x. 10.1186/s12866-019-1548-x PMC6712873 31455230

[11] Ministry of Agriculture and Rural Affairs of the People’s Republic of China. Nutrient requirements of meat-type sheep and goat: NY/T 816–2021. Beijing: China Agriculture Press; 2021.

[12] Ramos-Morales E, Arco-Pérez A, Martín-García AL, Yáñez-Ruiz DR, Frutos P, Hervás G. Use of stomach tubing as an alternative to rumen cannulation to study ruminal fermentation and microbiota in sheep and goats. Anim Feed Sci Technol. 2014;198:57–66. 10.1016/j.anifeedsci.2014.09.016.

[13] Jia XW, Zhang YX, Tian BY, Zhang GJ, Mao SY, Qian WX, et al. Integrative analysis of rumen microbiota and host multi-organ interactions underlying feed conversion efficiency in Hu sheep. J Anim Sci Biotechnol. 2026;17:19. 10.1186/s40104-025-01333-3. 10.1186/s40104-025-01333-3 PMC12865948 41630069

[14] Yin DF, Zhang Z, Zhu YL, Xu Z, Liu WQ, Liang K, et al. Assessment of the impact of dietary supplementation with epigallocatechin gallate (EGCG) on antioxidant status, immune response, and intestinal microbiota in post-weaning rabbits. Animals. 2024;14(20):3011. 10.3390/ani14203011. 39457941 10.3390/ani14203011 PMC11504044

[15] Li S, Zeng H, Wang C, Han Z. Effect of methionine hydroxy analog on hu sheep digestibility, rumen fermentation, and rumen microbial community in vitro. Metabolites. 2023;13(2):169. 10.3390/metabo13020169. 10.3390/metabo13020169 PMC9968006 36837788

[16] Ge T, Yang C, Li B, Huang XY, Zhao LY, Zhang XQ, et al. High-energy diet modify rumen microbial composition and microbial energy metabolism pattern in fattening sheep. BMC Vet Res. 2023;19:32. 10.1186/s12917-023-03592-6. 10.1186/s12917-023-03592-6 PMC9893671 36732756

[17] Liu X, Sha YZ, Dingkao RQ, Zhang W, Lv WB, Wei H, et al. Interactions between rumen microbes, VFAs, and host genes regulate nutrient absorption and epithelial barrier function during cold season nutritional stress in tibetan sheep. Front Microbiol. 2020;11:593062. 10.3389/fmicb.2020.593062. 33250882 10.3389/fmicb.2020.593062 PMC7674685

[18] Wang JL, Zhang T, Shen XT, Liu J, Zhao DL, Sun YW, et al. Serum metabolomics for early diagnosis of esophageal squamous cell carcinoma by UHPLC-QTOF/MS. Metabolomics. 2016;12(7):116. 10.1007/s11306-016-1050-5.

[19] Zhou ZW, Luo MD, Zhang HS, Yin YD, Cai YP, Zhu ZJ. Metabolite annotation from knowns to unknowns through knowledge-guided multi-layer metabolic networking. Nat Commun. 2022;13:6656. 10.1038/s41467-022-34537-6. 10.1038/s41467-022-34537-6 PMC9636193 36333358

[20] Zhang RY, Liu JH, Jiang LS, Wang XF, Mao SY. The remodeling effects of high-concentrate diets on microbial composition and function in the hindgut of dairy cows. Front Nutr. 2022;8:809406. 10.3389/fnut.2021.809406. 35178417 10.3389/fnut.2021.809406 PMC8845480

[21] Cheng AC, Peng XF, Chen WZ, Tseng DY, Tan ZG, Liu HJ, et al. Dietary probiotic *Aspergillus niger* preparation improves the growth performance, health status, and gut microbiota of white shrimp, *Penaeus vannamei*. Aquaculture. 2023;577:739988. 10.1016/j.aquaculture.2023.739988.

[22] Pittaluga A, Relling AE. Inclusion of a multispecies fungal feed additive in forage-based diets fed to beef cattle: Effects on growth performance and ruminal fermentation. J Anim Sci. 2024;102(Suppl 2):214–5. 10.1093/jas/skae102.243.

[23] Wang LJ, Lv ZQ, Ning XD, Yue ZG, Wang P, Liu CQ, et al. The effects of compound probiotics on production performance, rumen fermentation and microbiota of *Hu* sheep. Front Vet Sci. 2024;11:1440432. 10.3389/fvets.2024.1440432. 39545259 10.3389/fvets.2024.1440432 PMC11560882

[24] Busher JT. Serum albumin and globulin. Clinical Methods: The History, Physical, and Laboratory Examinations. 1990;3:497–9. 21250045

[25] Wei JT, Guo WZ, Yang XH, Chen F, Diao QY. Effects of dietary ramie level on growth performance, serum biochemical indices, and meat quality of Boer goats. Trop Anim Health Prod. 2019;51(7):1935–41. 10.1007/s11250-019-01891-5. 31134555 10.1007/s11250-019-01891-5

[26] Abou-Seri HS, Mahmoud S. The influence of *Aspergillus oryzae* fermentation product either alone or with malate salt supplementation to dairy buffalo cows on some blood traits and reproductive performance during transition period. J Egypt Vet Med. 2022;82(4):209–27.

[27] Kumar A, Pramanikt AK, Mandal TK. Immunological and haemobiochemical changes induced by oxytetraycline in Black Bengal goats. Indian J Vet Med. 2012;32:1–5.

[28] Ighodaro OM, Akinloye OA. First line defence antioxidants-superoxide dismutase (SOD), catalase (CAT) and glutathione peroxidase (GPX): their fundamental role in the entire antioxidant defence grid. Alexandria J Med. 2018;54(4):287–93. 10.1016/j.ajme.2017.09.001.

[29] Wang JG, Fu SQ, Yin XH, Sun SQ, Gao TY. Effects of *Aspergillus niger* and its compound preparations on methane emissions and gastrointestinal microbiota in heat-stressed holstein bulls. Animals. 2026;16(2):154. 10.3390/ani16020154. 41594344 10.3390/ani16020154 PMC12837928

[30] Williams CL, Thomas BJ, McEwan NR, Rees Stevens P, Creevey CJ, Huws SA. Rumen protozoa play a significant role in fungal predation and plant carbohydrate breakdown. Front Microbiol. 2020;11:720. 10.3389/fmicb.2020.00720. 32411103 10.3389/fmicb.2020.00720 PMC7200989

[31] Hespell RB. The genera *Succinivibrio* and *Succinimonas*. In: Balows A, Trüper HG, Dworkin M, Harder W, Schleifer KH, editors. The Prokaryotes. New York, NY: Springer. 1992:3979–3982. 10.1007/978-1-4757-2191-1_60.

[32] Alaa MA, Zhang H, Duan HW, Zhang JY, Mao SY. Metagenomic analysis reveals rumen microbiome enrichment and functional genes adjustment in carbohydrate metabolism induced by different sorting behavior in mid-lactation dairy cows. Anim Microbiome. 2025;7:82. 10.1186/s42523-025-00439-3. 10.1186/s42523-025-00439-3 PMC12302734 40722120

[33] Mizrahi I, Wallace RJ, Moraïs S. The rumen microbiome: balancing food security and environmental impacts. Nat Rev Microbiol. 2021;19(9):553–66. 10.1038/s41579-021-00543-6. 33981031 10.1038/s41579-021-00543-6

[34] Dao TK, Do TH, Le NG, Nguyen HD, Nguyen TQ, Le TTH, et al. Understanding the role of *Prevotella* genus in the digestion of lignocellulose and other substrates in Vietnamese native goats’ rumen by metagenomic deep sequencing. Animals. 2021;11(11):3257. 10.3390/ani11113257. 34827987 10.3390/ani11113257 PMC8614338

[35] Kumar S, Altermann E, Leahy SC, Jauregui R, Jonker A, Henderson G, et al. Genomic insights into the physiology of *Quinella*, an iconic uncultured rumen bacterium. Nat Commun. 2022;13:6240. 10.1038/s41467-022-34013-1. 10.1038/s41467-022-34013-1 PMC9585023 36266280

[36] Abrão FO, Duarte ER, Pessoa MS, Santos VL, Freitas Júnior LF, Barros KO, et al. Notable fibrolytic enzyme production by *Aspergillus* spp. isolates from the gastrointestinal tract of beef cattle fed in lignified pastures. PLoS ONE. 2017;12(8):e0183628. 10.1371/journal.pone.0183628. 28850605 10.1371/journal.pone.0183628 PMC5574564

[37] Kittelmann S, Pinares-Patino CS, Seedorf H, Kirk MR, Ganesh S, McEwan JC, et al. Two different bacterial community types are linked with the low-methane emission trait in sheep. PLoS ONE. 2014;9(7):e103171. 10.1371/journal.pone.0103171. 25078564 10.1371/journal.pone.0103171 PMC4117531

[38] Wang W, Wei ZY, Li ZH, Ren JR, Song YL, Xu JY, et al. Integrating genome-and transcriptome-wide association studies to uncover the host-microbiome interactions in bovine rumen methanogenesis. Imeta. 2024;3(5):e234. 10.1002/imt2.234. 39429883 10.1002/imt2.234 PMC11487568

[39] Kowalik B, Skomiał J, Pająk JJ, Taciak M, Majewska M, Bełżecki G. Population of ciliates, rumen fermentation indicators and biochemical parameters of blood serum in heifers fed diets supplemented with yeast (*Saccharomyces cerevisiae*) preparation. Anim Sci Pap Rep. 2012;30(4):329–38.

[40] Riyanti L, Evvyernie D. *In vitro* fermentation characteristics and rumen microbial population of diet supplemented with *Saccharomyces cerevisiae* and rumen microbe probiotics. Media Peternakan. 2016;39(1):40. 10.5398/medpet.2016.39.1.40.

[41] Franzolin R, Dehority BA. The role of pH on the survival of rumen protozoa in steers. Rev Bras Zootecn. 2010;39(10):2262–7. 10.1590/s1516-35982010001000023.

[42] Dehority BA. Effect of pH on viability of *Entodinium caudatum*, *Entodinium exiguum*, *Epidinium caudatum*, and *Ophryoscolex purkynjei in vitro*. J Eukaryot Microbiol. 2005;52(4):339–42. 10.1111/j.1550-7408.2005.00041.x. 16014011 10.1111/j.1550-7408.2005.00041.x

[43] Ortega Cerrilla ME, Mendoza Martínez G. Starch digestion and glucose metabolism in the ruminant: a review. Interciencia. 2003;28(7):380–6.

[44] Bohra V, Dafale NA, Purohit HJ. Understanding the alteration in rumen microbiome and cazymes profile with diet and host through comparative metagenomic approach. Arch Microbiol. 2019;201(10):1385–97. 10.1007/s00203-019-01706-z. 31338542 10.1007/s00203-019-01706-z

[45] Chu YD, Hao ZZ, Wang KK, Tu T, Huang HQ, Wang Y, et al. The GH10 and GH48 dual-functional catalytic domains from a multimodular glycoside hydrolase synergize in hydrolyzing both cellulose and xylan. Biotechnol Biofuels. 2019;12(1):279. 10.1186/s13068-019-1617-2. 31827607 10.1186/s13068-019-1617-2 PMC6892212

[46] Sidar A, Albuquerque ED, Voshol GP, Ram AFJ, Vijgenboom E, Punt PJ. Carbohydrate binding modules: diversity of domain architecture in amylases and cellulases from filamentous microorganisms. Front Bioeng Biotechnol. 2020;8:871. 10.3389/fbioe.2020.00871. 32850729 10.3389/fbioe.2020.00871 PMC7410926

[47] Grishutin SG, Gusakov AV, Markov AV, Ustinov BB, Semenova MV, Sinitsyn AP. Specific xyloglucanases as a new class of polysaccharide-degrading enzymes. Biochimica et Biophysica Acta (BBA) - General Subjects. 2004;1674(3):268–81. 10.1016/j.bbagen.2004.07.001. 15541296 10.1016/j.bbagen.2004.07.001

[48] Attia M, Stepper J, Davies GJ, Brumer H. Functional and structural characterization of a potent GH 74 *endo*-xyloglucanase from the soil saprophyte *Cellvibrio japonicus* unravels the first step of xyloglucan degradation. FEBS J. 2016;283(9):1701–19. 10.1111/febs.13696. 26929175 10.1111/febs.13696

[49] Ohsumi Y. Historical landmarks of autophagy research. Cell Res. 2014;24(1):9–23. 10.1038/cr.2013.169. 24366340 10.1038/cr.2013.169 PMC3879711

[50] Majerus PW, Ross TS, Cunningham TW, Caldwell KK, Jefferson AB, Bansal VS. Recent insights in phosphatidylinositol signaling. Cell. 1990;63(3):459–65. 10.1016/0092-8674(90)90442-h. 2225061 10.1016/0092-8674(90)90442-h

[51] Cui RR, Mao DA, Yi L, Wang C, Zhang XX, Xie H, et al. Apelin suppresses apoptosis of human vascular smooth muscle cells via APJ/PI3-K/Akt signaling pathways. Amino Acids. 2010;39(5):1193–200. 10.1007/s00726-010-0555-x. 20495838 10.1007/s00726-010-0555-x

[52] Diggle SP, Crusz SA, Cámara M. Quorum sensing. Curr Biol. 2007;17(21):R907–10. 10.1016/j.cub.2007.08.045. 17983563 10.1016/j.cub.2007.08.045

[53] Wang JD, Levin PA. Metabolism, cell growth and the bacterial cell cycle. Nat Rev Microbiol. 2009;7(11):822–7. 10.1038/nrmicro2202. 19806155 10.1038/nrmicro2202 PMC2887316

[54] Cao HL, Bougouffa S, Park TJ, Lau A, Tong MK, Chow KH, et al. Sharing of antimicrobial resistance genes between humans and food animals. mSystems. 2022;7(6):e00775–822. 10.1128/msystems.00775-22. 36218363 10.1128/msystems.00775-22 PMC9765467

[55] Sun J, Chen C, Cui CY, Zhang Y, Liu X, Cui ZH, et al. Plasmid-encoded *tet* (X) genes that confer high-level tigecycline resistance in *Escherichia coli*. Nat Microbiol. 2019;4(9):1457–64. 10.1038/s41564-019-0496-4. 31235960 10.1038/s41564-019-0496-4 PMC6707864

[56] Ma T, Zaheer R, McAllister TA, Guo W, Li FY, Tu Y, et al. Expressions of resistome is linked to the key functions and stability of active rumen microbiome. Anim Microbiome. 2022;4:38. 10.1186/s42523-022-00189-6. 10.1186/s42523-022-00189-6 PMC9167530 35659381

[57] Fitzpatrick D, Walsh F. Antibiotic resistance genes across a wide variety of metagenomes. FEMS Microbiol Ecol. 2016;92(2):fiv168. 10.1093/femsec/fiv168. 26738556 10.1093/femsec/fiv168

[58] Zhang AN, Gaston JM, Dai CZL, Zhao SJ, Poyet M, Groussin M, et al. An omics-based framework for assessing the health risk of antimicrobial resistance genes. Nat Commun. 2021;12:4765. 10.1038/s41467-021-25096-3. 10.1038/s41467-021-25096-3 PMC8346589 34362925

[59] Grutsch AA, Nimmer PS, Pittsley RH, Mckillip JL. *Bacillus* spp. as pathogens in the dairy industry. Foodborne Dis. 2018:193–211. 10.1016/b978-0-12-811444-5.00007-5.

[60] Li YY, Li C, Aqeel SM, Wang YC, Zhang Q, Ma JN, et al. Enhanced expression of xylanase in *Aspergillus niger* enabling a two-step enzymatic pathway for extracting β-glucan from oat bran. Bioresour Technol. 2023;377:128962. 10.1016/j.biortech.2023.128962. 36966944 10.1016/j.biortech.2023.128962

[61] Conway BAD, Venu V, Speert DP. Biofilm formation and acyl homoserine lactone production in the *Burkholderia cepacia* complex. J Bacteriol. 2002;184(20):5678–85. 10.1128/jb.184.20.5678-5685.2002. 12270826 10.1128/JB.184.20.5678-5685.2002 PMC139610

[62] Xie XM, Yang H, Zhao XG, Teng L, Yang YZ, Luo HL. Potential role of key rumen microbes in regulating host health and growth performance in Hu sheep. Anim Microbiome. 2025;7:51. 10.1186/s42523-025-00412-0. 10.1186/s42523-025-00412-0 PMC12103811 40414888

[63] Rasmussen TB, Givskov M. Quorum-sensing inhibitors as anti-pathogenic drugs. Int J Med Microbiol. 2006;296(2–3):149–61. 10.1016/j.ijmm.2006.02.005. 16503194 10.1016/j.ijmm.2006.02.005

[64] Xu YB, Wang YY, Ding XQ, Wang JS, Zhan XA. Inhibitory effects of reuterin on biofilm formation, quorum sensing and virulence genes of *Clostridium perfringens*. LWT-Food Sci Technol. 2022;162:113421. 10.1016/j.lwt.2022.113421.

[65] Ma YH, Elmhadi M, Wang C, Zhang H, Wang HR. Dietary supplementation of thiamine down-regulates the expression of mitophagy and endoplasmic reticulum stress-related genes in the rumen epithelium of goats during high-concentrate diet feeding. Ital J Anim Sci. 2021;20(1):2220–31. 10.1080/1828051X.2021.1985944.

[66] Schwab EC, Schwab CG, Shaver RD, Girard CL, Putnam DE, Whitehouse NL. Dietary forage and nonfiber carbohydrate contents influence B-vitamin intake, duodenal flow, and apparent ruminal synthesis in lactating dairy cows. J Dairy Sci. 2006;89(1):174–87. 10.3168/jds.S0022-0302(06)72082-3. 16357281 10.3168/jds.S0022-0302(06)72082-3

[67] Pan XH, Yang L, Beckers Y, Xue FG, Tang ZW, Jiang LS, et al. Thiamine supplementation facilitates thiamine transporter expression in the rumen epithelium and attenuates high-grain-induced inflammation in low-yielding dairy cows. J Dairy Sci. 2017;100(7):5329–42. 10.3168/jds.2016-11966. 28501402 10.3168/jds.2016-11966

[68] Phuntsok T, Froetschel MA, Amos HE, Zheng M, Huang YW. Biogenic amines in silage, apparent postruminal passage, and the relationship between biogenic amines and digestive function and intake by steers. J Dairy Sci. 1998;81(8):2193–203. 10.3168/jds.S0022-0302(98)75798-4. 9749385 10.3168/jds.S0022-0302(98)75798-4

[69] Sun XD, Yuan X, Chen L, Wang TT, Wang Z, Sun GQ, et al. Histamine induces bovine rumen epithelial cell inflammatory response *via* NF-κB pathway. Cell Physiol Biochem. 2017;42(3):1109–19. 10.1159/000478765. 28668950 10.1159/000478765

[70] Wu SJ, Gao SJ, Lin D, Bekhit AEA, Chen Y. Intestinal barrier restoration in UC: dietary protein/peptide mediate microbiota-Trp-AhR axis and food processing implications. Food Res Int. 2025;217:116799. 10.1016/j.foodres.2025.116799. 40597515 10.1016/j.foodres.2025.116799

[71] Venkatesh M, Mukherjee S, Wang HW, Li H, Sun K, Benechet AP, et al. Symbiotic bacterial metabolites regulate gastrointestinal barrier function via the xenobiotic sensor PXR and toll-like receptor 4. Immunity. 2014;41(2):296–310. 10.1016/j.immuni.2014.06.014. 25065623 10.1016/j.immuni.2014.06.014 PMC4142105

[72] Yang J, Wang H, Yan JA, Sun J, Wang YY, Huang GG, et al. Biotherapeutic potential of gut microbiota-derived indole-3-acetic acid. Crit Rev Microbiol. 2025;52(1):118–38. 10.1080/1040841x.2025.2532611. 40665738 10.1080/1040841X.2025.2532611

[73] Wu PY, Yao SY, Wang X, Yang L, Wang SL, Dai WB, et al. Oral administration of nanoformulated indoximod ameliorates ulcerative colitis by promoting mitochondrial function and mucosal healing. Int J Pharm. 2023;637:122813. 10.1016/j.ijpharm.2023.122813. 36905975 10.1016/j.ijpharm.2023.122813

[74] Coates MD, Tekin I, Vrana KE, Mawe GM. The many potential roles of intestinal serotonin (5-hydroxytryptamine, 5-HT) signalling in inflammatory bowel disease. Aliment Pharmacol Ther. 2017;46(6):569–80. 10.1111/apt.14226. 28737264 10.1111/apt.14226

[75] Chen Q, Zhang K, Jiao MJ, Jiao JK, Chen DL, Yin YH, et al. Study on the mechanism of mesaconitine-induced hepatotoxicity in rats based on metabonomics and toxicology network. Toxins. 2022;14(7):486. 10.3390/toxins14070486. 35878224 10.3390/toxins14070486 PMC9322933

