# Supplementary material for: A preliminary metagenomic and metabolomic investigation into the effects of Aspergillus niger cultures on microbial homeostasis and antibiotic resistance gene profiles in the rumen of fattening sheep
Source: J Anim Sci Biotechnol. 2026 May 26;17:100. doi: 10.1186/s40104-026-01412-z (PMC13202773; doi:10.1186/s40104-026-01412-z)
Supplement: Supplementary file 1 — Additional file 1: Table S1. Ingredients and chemical composition of the basal diets. [file 40104_2026_1412_MOESM1_ESM.docx]

**Table S1** Ingredients and chemical composition of the basal diets.

| **Ingredients, % of DM^1^** |  | **Chemical composition, % of DM** |  |
| --- | --- | --- | --- |
| Alfalfa | 15 | CP | 14.33 |
| Peanut vine | 15 | EE | 3.86 |
| Corn | 47 | NDF | 25.06 |
| Soybean meal | 10.7 | ADF | 15.61 |
| Wheat bran | 6 | Ca% | 0.99 |
| Corn germ meal | 3 | P% | 0.51 |
| Salt | 0.5 | ME（MJ/kg） | 10.18 |
| Stone powder | 1 |  |  |
| Dicalcium phosphate | 0.8 |  |  |
| Premix^2^ | 1.00 |  |  |

^1^DM: dry matter; CP, crude protein; EE, ether extract; NDF, neutral detergent fiber; ADF, acid detergent fiber; ME, metabolizable energy.

^2^Premix: Mineral content per kg of premix: VA 15000IU, VD 2200IU, VE 50IU, Fe 55mg, Cu 12.5mg, Mn 47mg, Zn 24mg, Se 0.5mg, I 0.5mg, Co 0.1mg.
