# Supplementary material for: A preliminary metagenomic and metabolomic investigation into the effects of Aspergillus niger cultures on microbial homeostasis and antibiotic resistance gene profiles in the rumen of fattening sheep
Source: J Anim Sci Biotechnol. 2026 May 26;17:100. doi: 10.1186/s40104-026-01412-z (PMC13202773; doi:10.1186/s40104-026-01412-z)
Supplement: Supplementary file 2 — Additional file 2: Fig. S1. Principal coordinates analysis (PCoA) and permutational multivariate analysis of variance (PERMANOVA) of carbohydrate active enzymes (CAZymes) (A) and Kyoto encyclopedia of genes and genomes (KEGG) level 3 (B), and the effects of dietary treatments on the relative abundance of rumen microbial CAZymes at the class (C) and family (D) levels, microbial categories at the KEGG level 2 (E), and KEGG level 3 (F). Fig. S2. Effects of dietary treatments on the relative abundance of rumen antibiotics resistance genes (ARGs) at the type level (A) and subtype level (B). Fig. S3. Effects of dietary treatments on functional contribution of tetracycline resistance genes in rumen at the phylum level (A), genus level (B) and species level (C), and on that of β-lactam resistance genes in rumen at the phylum level (D), genus level (E) and species level (F). Fig. S4. Effects of dietary treatments on differences in rumen microbial metabolites by partial least squares discriminant analysis (PLS-DA) (A) and orthogonal partial least squares discriminant analysis (OPLS-DA) (B). [file 40104_2026_1412_MOESM2_ESM.docx]

**
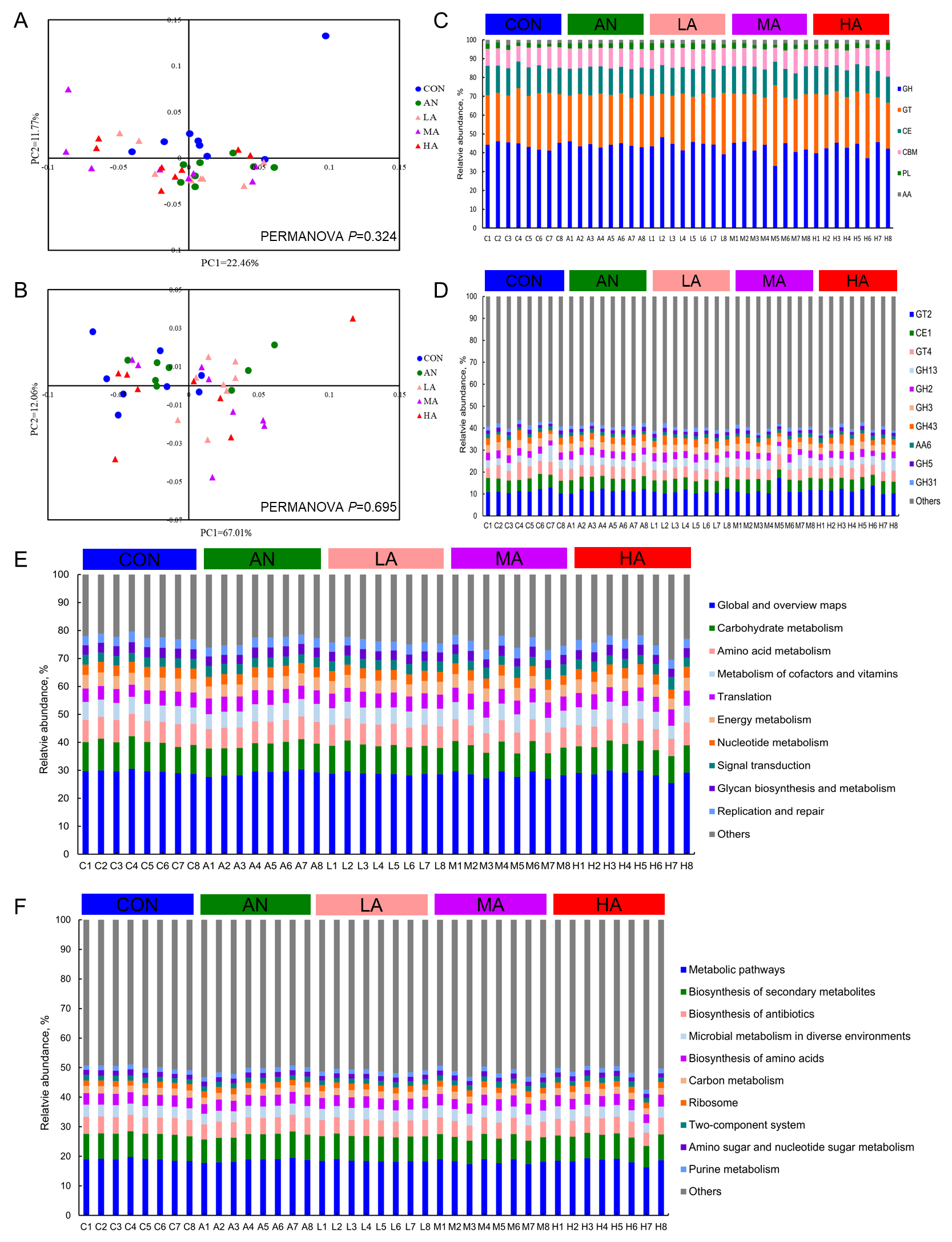
Fig. S1** Principal coordinates analysis (PCoA) and permutational multivariate analysis of variance (PERMANOVA) of carbohydrate active enzymes (CAZymes) (**A**) and Kyoto encyclopedia of genes and genomes (KEGG) level 3 (**B**), and the effects of dietary treatments on the relative abundance of rumen microbial CAZymes at the class (**C**) and family (**D**) levels, microbial categories at the KEGG level 2 (**E**), and KEGG level 3 (**F**). CON = a control diet; AN = a control diet + 5000 mg/kg chlortetracycline premix; LA = a control diet + 250 mg/kg *A. niger* cultures; MA = a control diet + 500 mg/kg *A. niger* cultures; HA = a control diet + 1000 mg/kg *A. niger* cultures.

**
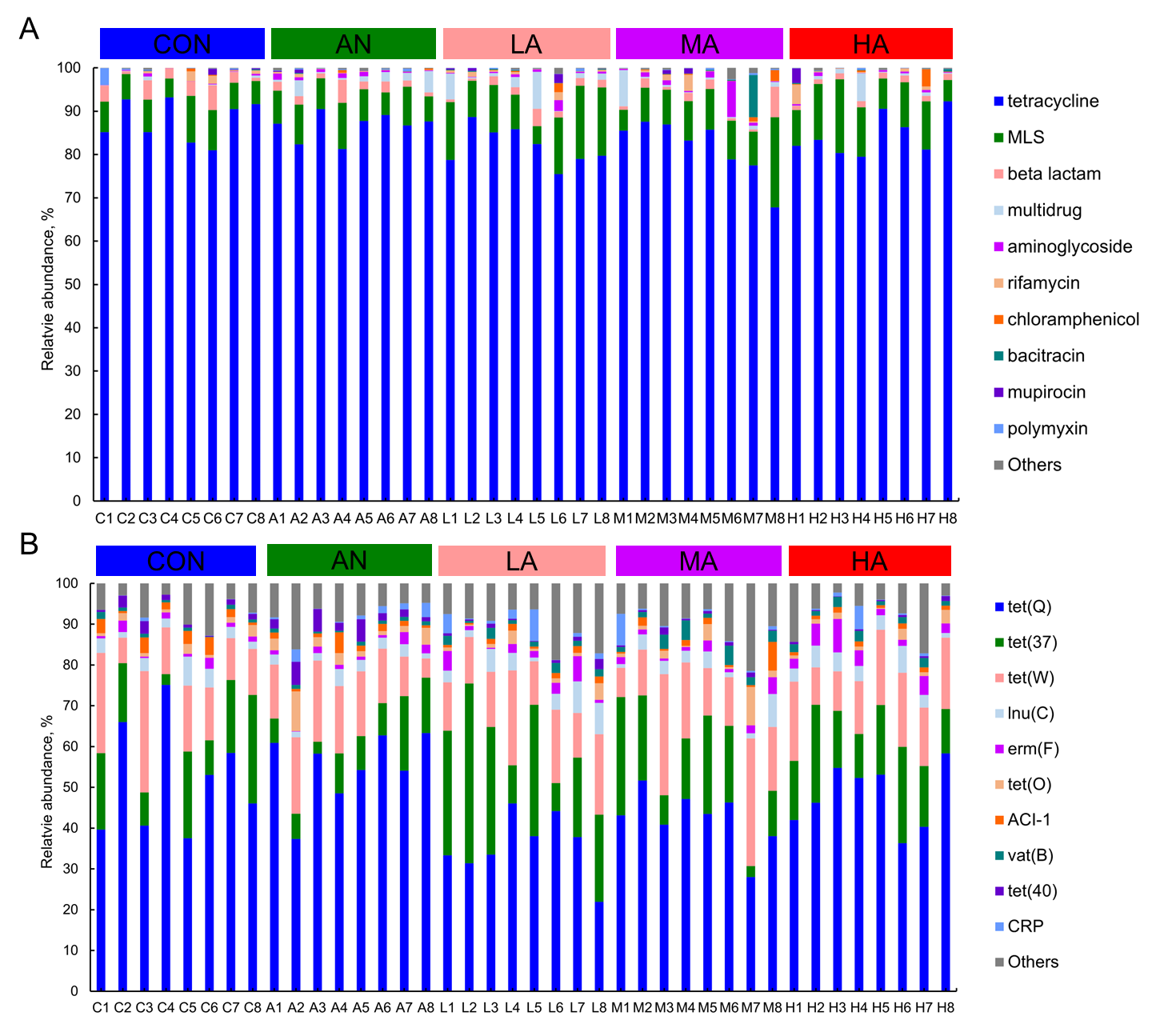
Fig. S2** Effects of dietary treatments on the relative abundance of rumen antibiotics resistance genes (ARGs) at the type level (**A**) and subtype level (**B**). CON = a control diet; AN = a control diet + 5000 mg/kg chlortetracycline premix; LA = a control diet + 250 mg/kg *A. niger* cultures; MA = a control diet + 500 mg/kg *A. niger* cultures; HA = a control diet + 1000 mg/kg *A. niger* cultures.


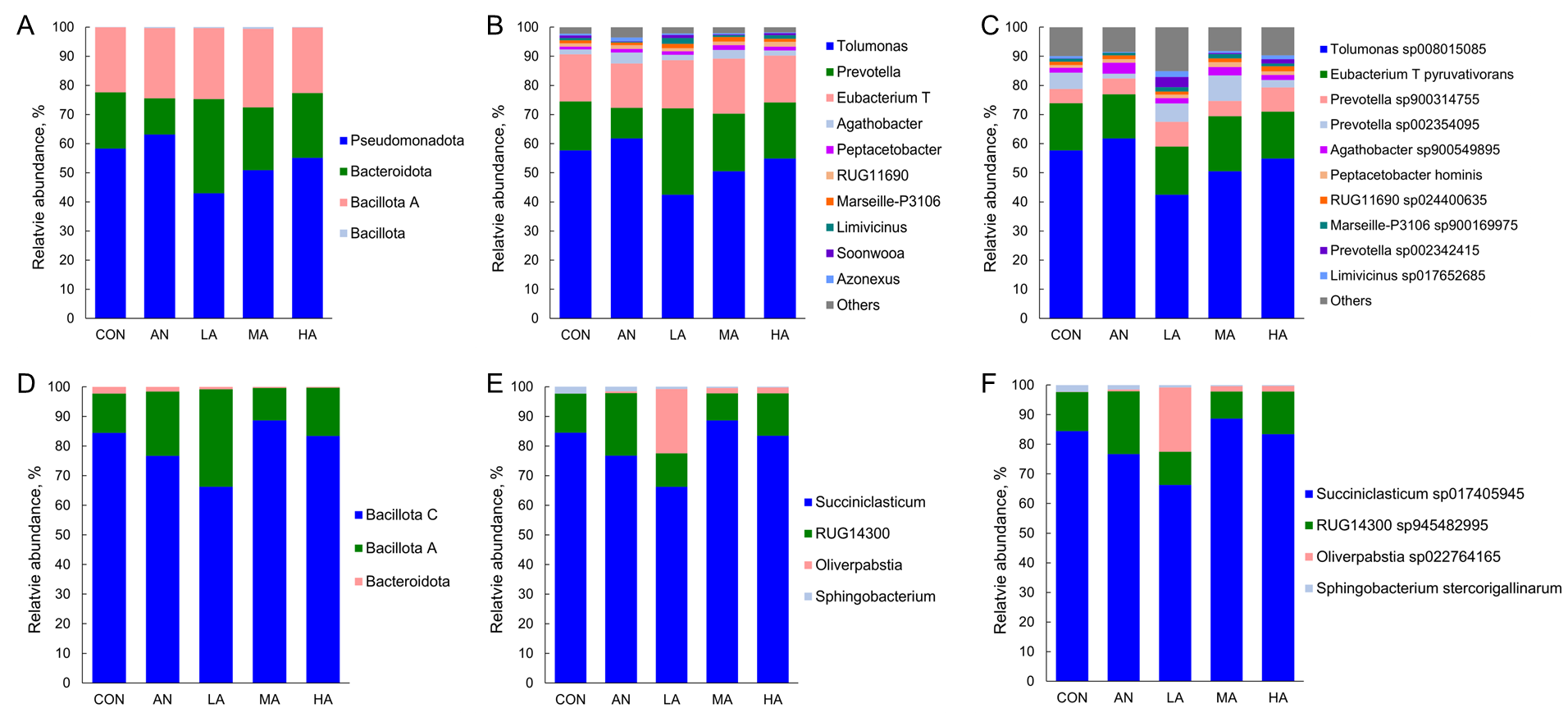
**Fig. S3** Effects of dietary treatments on functional contribution of tetracycline resistance genes in rumen at the phylum level (**A**), genus level (**B**) and species level (**C**), and on that of β-lactam resistance genes in rumen at the phylum level (**D**), genus level (**E**) and species level (**F**). CON = a control diet; AN = a control diet + 5000 mg/kg chlortetracycline premix; LA = a control diet + 250 mg/kg *A. niger* cultures; MA = a control diet + 500 mg/kg *A. niger* cultures; HA = a control diet + 1000 mg/kg *A. niger* cultures.


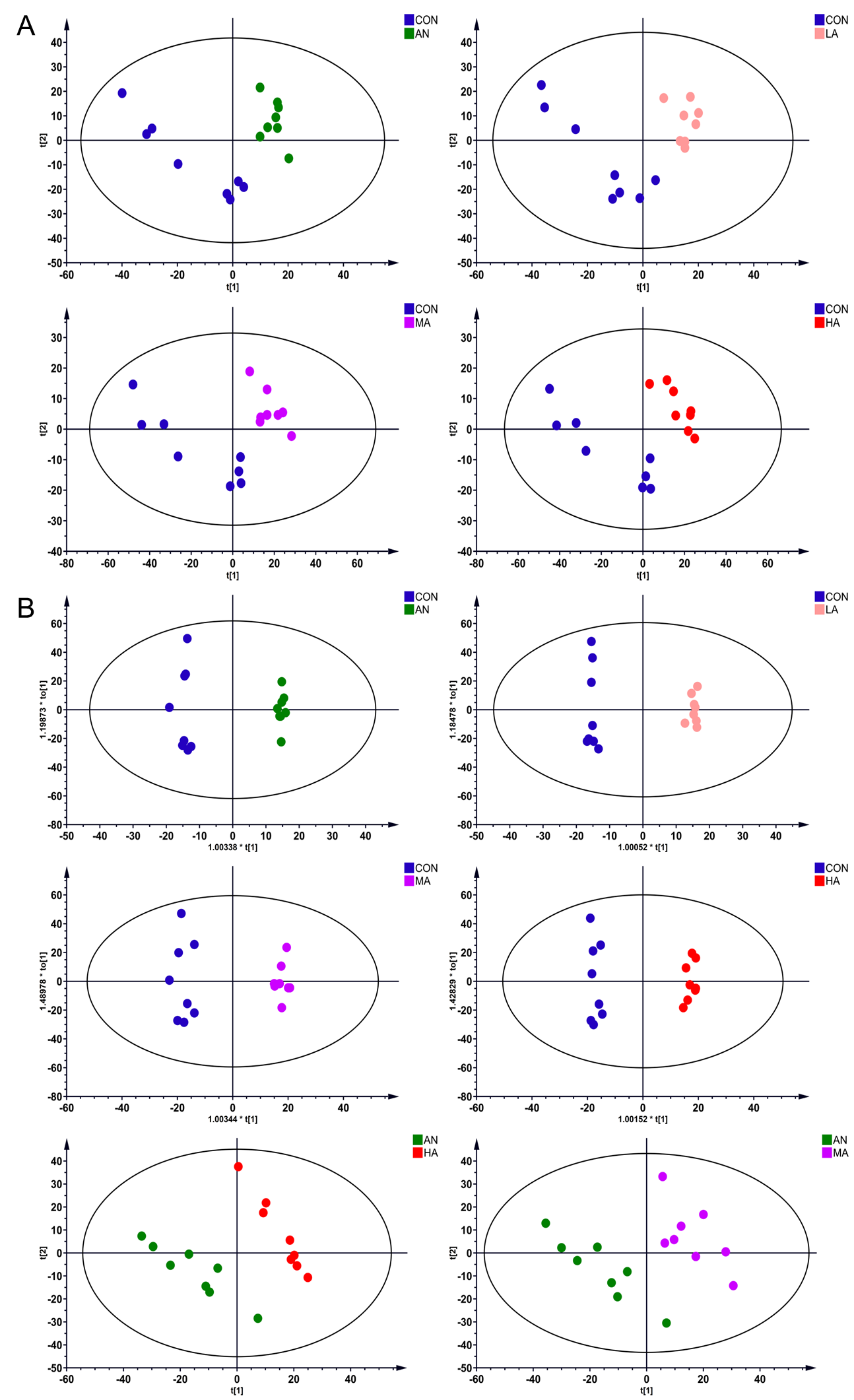


**Fig. S4** Effects of dietary treatments on differences in rumen microbial metabolites by partial least squares discriminant analysis (PLS-DA) (**A**) and orthogonal partial least squares discriminant analysis (OPLS-DA) (**B**). CON = a control diet; AN = a control diet + 5000 mg/kg chlortetracycline premix; LA = a control diet + 250 mg/kg *A. niger* cultures; MA = a control diet + 500 mg/kg *A. niger* cultures; HA = a control diet + 1000 mg/kg *A. niger* cultures.
